# Gut liver brain axis in diseases: the implications for therapeutic interventions

**DOI:** 10.1038/s41392-023-01673-4

**Published:** 2023-12-06

**Authors:** Mengyao Yan, Shuli Man, Benyue Sun, Long Ma, Lanping Guo, Luqi Huang, Wenyuan Gao

**Affiliations:** 1 State Key Laboratory of Food Nutrition and Safety, Key Laboratory of Industrial Microbiology, Ministry of Education, Tianjin Key Laboratory of Industry Microbiology, National and Local United Engineering Lab of Metabolic Control Fermentation Technology, China International Science and Technology Cooperation Base of Food Nutrition/Safety and Medicinal Chemistry, College of Biotechnology, Tianjin University of Science & Technology, 300457 Tianjin, China; 2 National Resource Center for Chinese Materia Medica, China Academy of Chinese Medical Sciences, 100700 Beijing, China; 3 Tianjin Key Laboratory for Modern Drug Delivery & High-Efficiency, School of Pharmaceutical Science and Technology, Tianjin University, Weijin Road, 300072 Tianjin, China

**Keywords:** Gastrointestinal diseases, Diseases of the nervous system, Endocrine system and metabolic diseases, Endocrine system and metabolic diseases

## Abstract

Gut-liver-brain axis is a three-way highway of information interaction system among the gastrointestinal tract, liver, and nervous systems. In the past few decades, breakthrough progress has been made in the gut liver brain axis, mainly through understanding its formation mechanism and increasing treatment strategies. In this review, we discuss various complex networks including barrier permeability, gut hormones, gut microbial metabolites, vagus nerve, neurotransmitters, immunity, brain toxic metabolites, β-amyloid (Aβ) metabolism, and epigenetic regulation in the gut-liver-brain axis. Some therapies containing antibiotics, probiotics, prebiotics, synbiotics, fecal microbiota transplantation (FMT), polyphenols, low FODMAP diet and nanotechnology application regulate the gut liver brain axis. Besides, some special treatments targeting gut-liver axis include farnesoid X receptor (FXR) agonists, takeda G protein-coupled receptor 5 (TGR5) agonists, glucagon-like peptide-1 (GLP-1) receptor antagonists and fibroblast growth factor 19 (FGF19) analogs. Targeting gut-brain axis embraces cognitive behavioral therapy (CBT), antidepressants and tryptophan metabolism-related therapies. Targeting liver-brain axis contains epigenetic regulation and Aβ metabolism-related therapies. In the future, a better understanding of gut-liver-brain axis interactions will promote the development of novel preventative strategies and the discovery of precise therapeutic targets in multiple diseases.

## Introduction

**Fig. 1:**
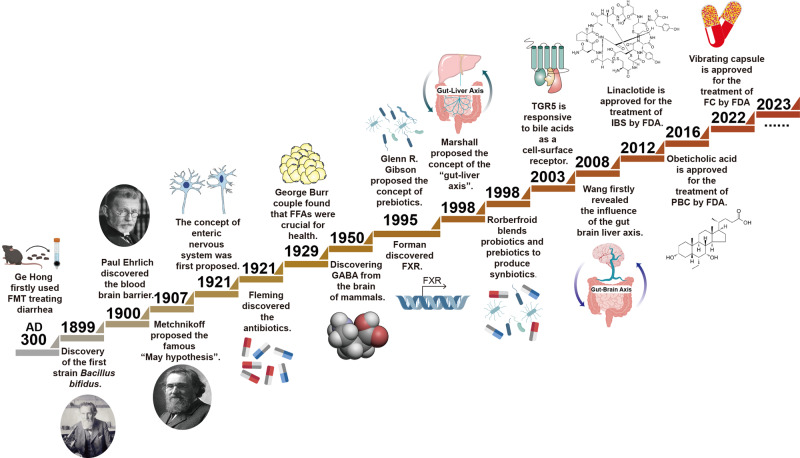
Timeline of the milestone events for the gut liver brain axis. In AD 300–400, Ge Hong collected folk remedies and published “Emergency Prescriptions for Elbow Reserve”, which firstly used fecal liquid to treat food poisoning and severe diarrhea. In 1899, Henry Tissier in France isolated the first strain of *Bacillus bifidus* from the feces of healthy breastfed infants. In 1900, German bacteriologist Paul Ehrlich discovered the blood–brain barrier. Metchnikoff proposed the famous “May hypothesis” in 1907, pointing out that the gut microbiota and its interactions with the host were crucial for health. In 1921, the concept of enteric nervous system (ENS) was first proposed, which focused on the neuroanatomy, function, and pathophysiology of gut-brain interactions. After six years, Wieland won the Nobel Prize in Chemistry for his discovery of bile acids and their chemical structures. In 1929, the George Burr couple discovered fatty acids were crucial for health. In 1950, gamma-aminobutyric acid (GABA) was discovered in the mammalian brain. In 1995, the concept of prebiotics was proposed by the international “father of prebiotics”, Glenn R. Gibson, and farnesoid X receptor (FXR) was first discovered by Forman et al. In 1998, Marshall proposed the concept of the “gut-liver axis”. Meanwhile, Rorberfroid further blended probiotics and prebiotics into products called synbiotics. In 2003, takeda G protein-coupled receptor 5 (TGR5) was first discovered as a cell surface receptor for bile acid reactions. In 2008, Wang first revealed the influence of the gut-brain-liver axis in human health. In 2012, the first gut-brain axis-related drug linaclotide was approved for the treatment of irritable bowel syndrome (IBS) by FDA. In 2016, the first gut-liver axis-related drug obeticholic acid was approved for the treatment of primary biliary cirrhosis by FDA. In 2022, the gut-brain axis-related drug vibrating capsule was approved for the treatment of functional constipation by FDA. Created with BioRender.com

In recent years, the importance of the liver brain axis in maintaining human health has received attention.^1^ Scientific investigations show that ties among gut dysbiosis or disruption, brain^2^ and liver^3^ diseases mean the pathophysiology of liver and brain diseases is frequently linked to gastrointestinal problems.^4,5^ For example, a leaky gut is described in nonalcoholic fatty liver disease (NAFLD),^6^ alcoholic liver disease (ALD),^7^ non-alcoholic steatohepatitis (NASH),^8^ alcoholic steatohepatitis (ASH),^9^ hepatocellular carcinoma (HCC),^10^ and so forth.^11^ Besides, gut dysbiosis is discovered in multiple gut brain axis-related diseases including Parkinson’s disease (PD),^12^ Alzheimer’s disease (AD),^13^ amyotrophic lateral sclerosis,^14^ autism,^15^ stroke,^16^ depression,^17^ and drug addiction.^18^ Some liver diseases are closely related to neurological disorders through liver-brain axis, such as hepatic encephalopathy (HE), cirrhosis, and so on. There are many milestone events for gut-liver-brain axis-related theory in the past few centuries. In AD 300–400, Ge Hong collected folk remedies and published “Emergency Prescriptions for Elbow Reserve” which first recorded fecal liquid treating food poisoning and severe diarrhea. In 1998, Marshall put forward the concept of the “gut liver axis”.^19^ After ten years, the influence of the gut liver brain axis in human health was first revealed.^20^ Therefore, the crosstalk among the gut, liver and brain is being increasingly recognized and delineated piece by piece (Fig. 1).^21^

Gut-liver brain axis is a three-way highway of communication.^22,23^ The connection between the gut and the liver lays on the gut barrier, whose disruption leads to more bacteria or their metabolites entering the liver^24,25^ and contributes to or worsens a variety of hepatic disorders.^26^ Various peptides or hormones produced by the intestines in response to nutrition influence neural signaling from the gut to the brain. They enter the blood, act on the local vagal, spinal afferent neurons and brain, then feeds back to the liver vagal parasympathetic nerves and innervates the gut and paracrine.^27^

There are multiple marketed drugs involved in the regulation of the gut-liver-brain axis (Table 1). For example, odevixibat is a pharmaceutical option for interfering with the enterohepatic circulation in individuals with progressive familial intrahepatic cholestasis.^28^ Vibrating capsule is a potential alternative physical treatment for functional constipation. It relieves gut burden, mental and physical stress.^29^ Besides, sodium oligomannate therapeutically remodels gut microbiota and neurological inflammation in AD development and is regarded as a unique technique for AD therapy via remodeling the gut-brain axis.^30^ In addition, some ongoing research on the gut-liver-brain axis is also constantly emerging. For example, because the microbiome controls intestinal permeability, and changes blood–brain barrier (BBB), vagus nerve, and neurotransmitters, the supplement of probiotics, prebiotics or synbiotics such as VSL#3,^31^ multistrain probiotics^32^ and galactooligosaccharides^33^ is regarded as an effective therapy strategy for the treatment of AFLD, NASH, autism spectrum disorder (ASD), depression, PD, schizophrenia, epilepsy, migraine, and so on.

**Table 1:** FDA approved drugs related to gut liver brain axis

Approval Date	Drugs	Disease	Mechanism	Locations
2012	Linaclotide	Irritable bowel syndrome	Altering guanylate cyclase^430,431^	USA
2016	Obeticholic acid	Primary biliary cirrhosis	Regulating gut-liver axis^432,433^	USA
2021	Odevixibat	Progressive familial intrahepatic cholestasis	Targeting IBAT^28,434^	USA
2022	Vibrating capsule	Functional constipation	Stimulating the ENS^29,435^	USA
2022	Sodium Oligomannate	AD	Regulating gut-brain axis^436,437^	China

The data is released from http://pharmdata.ncmi.cn/globaldrugs/index.asp

In this review, we discuss the comprehensive pathophysiology of the gut-liver-brain axis in several chronic liver diseases, nervous and gut disorders, and introduce the candidates now being explored in this axis. These findings have significant implications for society as well as broad health issues throughout the world, which urgently need to be addressed. It is expected to further develop more clinical candidates to regulate gut-liver-brain axis.

## Mechanisms linking the gut-liver axis

**Fig. 2:**
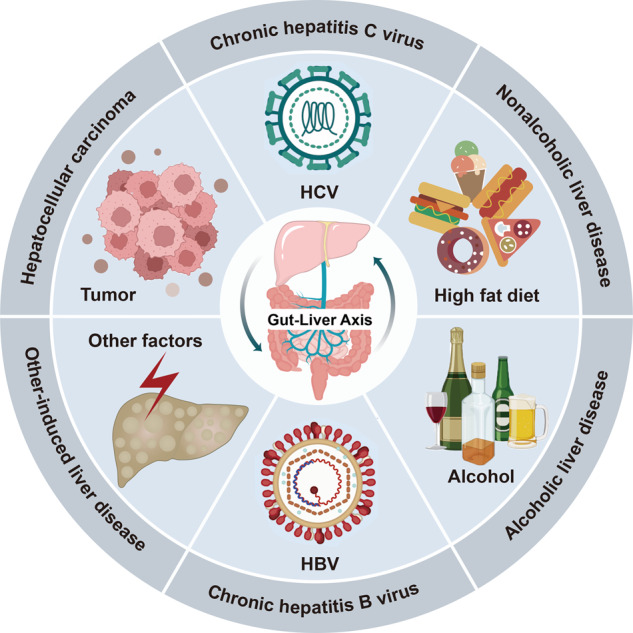
An outline map of various diseases in the gut-liver axis are currently implicated including chronic HBV, chronic HCV, NAFLD, ALD, other-induced liver disease, and HCC. Created with BioRender.com

**Fig. 3:**
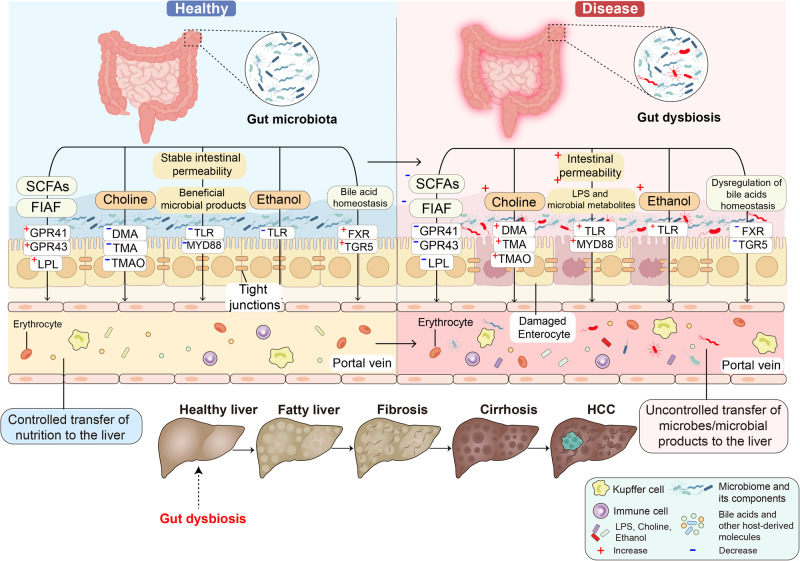
Gut dysbiosis influences liver disease progression. (1) Gut dysbiosis increases the number of pathogens and the release of their metabolites like lipopolysaccharide (LPS) and destroys tight junctions (TJs) and gut permeability. (2) Gut dysbiosis changes SCFAs and FIAF production. (3) Gut dysbiosis increases intestinal choline and ethanol production. (4) Gut dysbiosis influences BAs metabolism. These factors and metabolites together with dietary lipids result in liver steatosis, inflammation, and eventually, HCC. Created with BioRender.com

Gut dysbiosis is a medical condition that happens when there is a microbial imbalance in a person’s intestines. Gut microbiota or their metabolites improve or aggravate the progression of multiple liver diseases such as chronic hepatitis B virus (HBV),^34^ chronic hepatitis C virus (HCV),^35^ NAFLD,^36^ ALD,^37^ other-induced liver disease^38^ and HCC^39^ (Fig. 2) through several mechanisms,^40^ including changes in the intestinal permeability,^41^ short-chain fatty acids (SCFAs),^42^ long-chain fatty acids (LCFAs),^43^ fasting-induced adipocyte factor (FIAF), choline metabolism,^44,45^ ethanol production,^46^ and BAs metabolism^47,48^ (Fig. 3).

### Changing intestinal permeability in the gut-liver axis

#### Microbiome controls intestinal permeability in the gut-liver axis

**Fig. 4:**
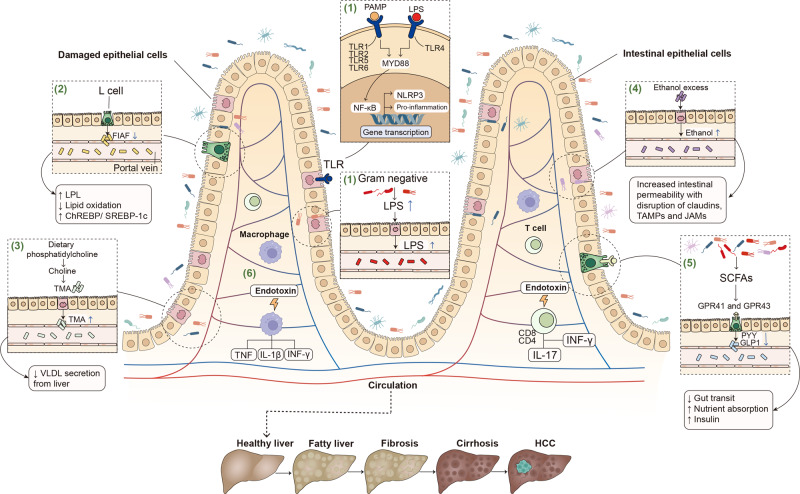
Gut microbiota and its metabolites in the gut-liver axis. (1) Microbial metabolites such as pathogen-associated molecular patterns (PAMP) and LPS bind to TLRs on the membrane of intestinal epithelial cells. Activation of these TLR/MYD88-dependent signaling pathways leads to the translocation of nuclear factor-kappa B (NF-κB) into the nucleus, and promotes the transcription of numerous cytokines. (2) Gut dysbiosis inhibits the secretion of FIAF, and then inhibits the release of endothelial LPL, which is responsible for the release of triglycerides from circulating chylomicrons and VLDL. (3) The intestinal microbiota converts dietary phosphatidylcholine to choline or hepatotoxic TMA. These metabolites increase intestinal permeability with disruption of TJs proteins such as claudins, TAMPs, and JAMs. (4) Gut dysbiosis also results in increased endogenous alcohol production, which allows endotoxins and ethanol directly into the liver. (5) Gut dysbiosis inhibits the secretion of SCFAs. It has effects on G-protein coupled receptors, such as GPR41 and GPR43, causing the release of PYY and GLP-1, respectively, from neuroendocrine L cells. (6) Endotoxins released from intestinal microbiota stimulate the secretion of inflammatory factors by immune cells. Created with BioRender.com

Gut microbiota communities are highly flexible, with their composition influenced by a variety of external and host factors such as high-fat diet, age, physiological condition, and genetic background.^49^ When gut dysbiosis happens, the amount of some gut microbiota including *Lactobacillus*, *Bacteroides,* and *Bifidobacterium* decreases, and leads to the damage of TJs protein^50^ and the change of intestinal permeability, which promotes the pathogens and their metabolites entering vessel circulation, and subsequently activates the proinflammatory pathways (Fig. 4).^51,52^ For example, intestinal microbial metabolites alter host gut mucosal proteins and lead to liver injury.^53^ Endogenous changes in the gut affect the intestinal barrier and promote intestinal inflammation.^54^ Besides, intestinal-associated lymphoid tissue participates in intestinal barrier function and prevents intestinal inflammation.^55^

#### Cytokines control intestinal permeability in the gut-liver axis

In general, the intestinal barrier consists of TJs proteins including transmembrane proteins such as claudins, TJs-associated marvel proteins (TAMPs), and junctional adhesion molecules (JAMs),^56,57^ and the scaffolding protein containing zona occludens (ZO)-1, ZO-2, and ZO-3.^58^ Cytokines control intestinal barrier function, especially tumor necrosis factor (TNF), interferon-gamma (IFN-γ), interleukin (IL)-1β, endotoxins and chemokines, which become the key mediators to destroy the intestinal barrier.^59^ Macrophages and T cells as the important immune cells maintain the balance of the barrier, whose position is close to the blood vessels.^60^ The activation of macrophages releases some inflammatory substances, which promote the development of steatosis, inflammation, and fibrosis.^61^

LPS as one of the pathogen-associated molecular patterns contains lipid A, crosses the intestinal mucosa via TJs or with the aid of chylomicrons, binds to the LPS binding protein (LBP), and interacts with toll-like receptor (TLR)/myeloid differentiation primary response 88 in the liver and gut mucosal tissues. It promotes the transcription of numerous cytokines in the liver and adipose tissues,^62^ the activation of liver inflammasomes via NACHT, LRR, and PYD domains-containing protein 3,^63^ and the development of fibrosis in the livers (Fig. 4).^64,65^ This may be linked to the increase of certain gram-negative bacterial genera, including *Bacteroides, Enterobacteria, Escherichia*, and *Proteus*, which is discovered in patients with NAFLD and NASH.^66^

### Orchestrating intestinal SCFAs, LCFAs, and FIAF in the gut-liver axis

#### SCFAs and LCFAs in the gut-liver axis

Normal gut microbiome everyday produces 50–100 mM of SCFAs such as acetic acid, propionic acid, butyric acid, and so forth.^67^ For example, butyric acid as the most important SCFAs is mainly generated by *Coprococcus*, *Faecalibacterium prausnitzii, Eubacterium rectale, Eubacterium hallii*, and *Roseburia bromii*.^68^ Acetic acid as the most productive SCFAs is mainly generated by gut bacteria, including *Prevotella*, *Ruminococcus*, *Bifidobacterium*, *Bacteroides*, *Clostridium*, *Streptococcus*, *Akkermansia muciniphila,* and *Hydrobacillus*.^69^ In addition, propionic acid as the third most important SCFAs is mainly synthesized by *Akkermansia municiphilla, Salmonella enterica serovar Typhimurium,* and *Roseburia inulinivorans*.^70^

SCFAs provide energy sources, and promote hepatic lipogenesis and gluconeogenesis via acting on the G-protein coupled receptors (GPR) such as GPR41 and GPR43.^71^ Butyrate is the most important SCFAs in sustaining colonic health since it directly provides energy to colonic epithelial cells.^72^ Almost all butyric acid is absorbed by colonocytes, while a small amount is distributed in peripheral blood.^73^ Butyrate directly acts on T regulatory cells in the mucosa, suppresses inflammation^74^ and fatty acid synthesis, and promotes the growth of probiotics.^75^ Acetate is absorbed by the proximal colon and swiftly transferred to the liver, where it acts as a substrate for cholesterol production.^76^ In addition, 90% of the third major SCFAs like propionic acid are delivered to the liver and used as a substrate for other pathways such as lipogenesis, gluconeogenesis and protein synthesis.^77^ All of the above SCFAs acting on GPR41 and GPR43 on L cells release peptide tyrosine tyrosine (PYY), which reduces gastric emptying and intestinal transit, and improves food absorption.^78^ These L cells also secrete glucagon-like peptide 1 (GLP-1) which promotes glucose-dependent insulin secretion.^79,80^

However, the impact of SCFAs is controversial.^81^ At present, the function of SCFAs in bacterial-host interactions is unclear. It is difficult to determine whether SCFAs are beneficial or harmful to the host.^82^ As a recent study, excessive accumulation of butyrate leads to cholestasis, hepatocyte mortality, and neutrophilic inflammatory reactions in the liver, which finally induces icteric HCC.^83^

LCFAs belonging to the derivatives of triglycerides are isolated from animal fats and vegetable oils and catalyzed by gut bacteria like *Lactobacillus rhamnosus GG*.^84^ For example, LCFAs favorably influence the bacterial population in the gut, which has been demonstrated to improve intestinal barrier function, decrease endotoxemia, and inhibit ALD.^85^ However, when LCFAs reach high concentration, they become toxic detergents within cells.^86^

#### FIAF in the gut-liver axis

In general, SCFAs especially for butyrate^87^ can further activate the release of FIAF (also named angiopoietin-related protein 4) from L cells, brown fat, white fat and hepatocytes (Fig. 4).^88^ Meanwhile, FIAF inhibits the expression of lipoprotein lipase (LPL)^6^ and triglyceride buildup in both adipose tissue and the livers.^88,89^ Inhibition of FIAF activates carbohydrate-responsive element-binding protein and sterol regulatory element-binding protein 1 in livers,^88,90^ which boosts lipogenic enzymes and increases fat formation.^91^

### Regulating intestinal production of choline and ethanol in the gut-liver axis

#### Choline metabolism in the gut-liver axis

Choline, a component of cell membranes, is generated endogenously in the liver^92^ and decomposed by gut bacteria (Fig. 4). Choline acts in the generation of very low-density lipoprotein (VLDL) because it is required for the formation of the phosphatidyl-choline component of VLDL particles in the liver.^93^ VLDL particles cannot be released in the deficiency of choline, which increases lipoperoxidation in hepatocytes, and in turn results in an increase in intracellular free radicals linked with DNA damage, apoptosis, and tumorigenesis. In the gut, the increased choline metabolism is closely related to high levels of the taxa *Firmicutes Erysipelotrichia*.^94^

Gut microbiota converts choline into dimethylamine and trimethylamine (TMA),^95,96^ and catalyzes choline or TMA into toxic metabolites like trimethylamine N-oxide (TMAO).^95^ L-carnitine, choline and betaine are the main substrates for TMA synthesis by gut bacterial strains including *Clostridium asparagiforme*, *Clostridium sporogenes*, *Clostridium hathewayi*, *Escherichia fergusonii*, *Anaerococcus hydrogenalis*, and *Proteus penneri*.^97^ Higher circulatory distribution of TMAO is associated with decreased levels of host-produced phosphatidylcholine, a sign of intestinal dysbiosis.^98^ This is related to liver damage as a result of increased triglyceride buildup, which causes hepatic steatosis.

#### Ethanol production in the gut-liver axis

Gut dysbiosis stimulates intestinal ethanol production, which is implicated in the development of NASH and NAFLD (Fig. 4).^99,100^ For example, 1 g of *Escherichia coli* generates 0.8 g of ethanol each hour in anaerobic conditions,^101^ further increases intestinal permeability and portal levels of LPS, triggers the expression of TLR and inflammasome, and contributes to liver injury. Ethanol significantly alters the composition of the gut microbiota, including decreasing the relative abundance of *Bacteroidetes* and increasing the relative abundance of *Proteobacteria*.^54^ Its metabolites, especially for acetaldehyde, may damage TJs of the gut epithelial tissue, cause a leaky gut, and facilitate bacterial and fungal translocation, which is related to the advancement of liver cirrhosis development.^102^

In addition, excessive alcohol in ALD damages intestinal barrier components, especially for proteins implicated in innate antibacterial defense such as 3-β and 3-γ, increases adhesion between bacteria and the mucosal surface, causes excessive growth of intestinal bacteria, microbial product translocation^103^ and ecological imbalances related to intestinal inflammation^101^ and liver inflammation.^104,105^

### Influencing BAs metabolism in the gut-liver axis

BAs are produced through cytochrome P450 enzymes (CYPs)-mediated oxidation of cholesterol, which includes the classical and alternative pathways in hepatocytes (Fig. 3).^106^ The classical process contains the enzymatic action of cholesterol 7 alpha-hydroxylase (CYP7A1), sterol 12α-hydroxylase (CYP8B1), and sterol 27-hydroxylase (CYP27A1) to generate the primary BAs such as cholic acid (CA) and chenodeoxycholic acid (CDCA). The alternative process includes CYP27A1 hydroxylating the cholesterol side chain to produce CDCA and then oxysterol 7α-hydroxylase (CYP7B1) 7-α hydroxylating to form the oxysterol intermediates.^107^

Subsequently, CA and CDCA conjugate taurine (primarily in mice) or glycine (primarily in humans) to form taurocholic acid (TCA), taurochenodeoxycholic acid (TCDCA), glycocholic acid (GCA) and glycochenodeoxycholic acid (GCDCA), respectively, and then are secreted from the liver into the gallbladder via the canalicular bile salt export pump (BSEP).^108^ In addition, some BAs undergo sulfonation and glucuronidation, and then are transported from the liver into the gallbladder via the multidrug resistance-associated protein 2.^109^

After BAs are synthesized in hepatocytes, they are secreted from the liver to the gallbladder and then into the intestine. Gut bacteria convert the primary BAs into secondary BAs.^110^ The main intestinal bacteria taking part in BAs metabolism include *Bacteroides*, *Clostridium*, *Lactobacillus*, *Bifidobacterium* and *Listeria* in BAs deconjugation,^111^ *Bacteroides*, *Eubacterium*, *Clostridium*, *Escherichia*, *Egghertella*, *Eubacterium*, *Peptostreptococcus,* and *Ruminococcus* in oxidation and epimerization of hydroxyl groups at C3, C7, and C12 of BAs, *Bacteroides*, *Eubacterium* and *Lactobacillus* in BAs esterification, and *Clostridium*, *Fusobacterium*, *Peptococcus,* and *Pesudomonas* in BAs desulfatation.^112^

In this process, intestinal anaerobes including genera *Bacteroides*, *Eubacterium* and *Clostridium* deconjugate taurine-conjugated BAs and glycine-conjugated BAs into unconjugated counterparts via microbiota metabolites like bile salt hydrolase. Subsequently, anaerobes containing the genera *Bacteroides*, *Clostridium*, *Eubacterium*, *Lactobacillus* and *Escherichia* convert these unconjugated primary BAs into the secondary BAs such as lithocholic acid (LCA) and deoxycholic acid (DCA) based on 7α-dehydroxylation of CYP7A1.^113^ Most of CA, CDCA and DCA are then reabsorbed in the gut and transported back to the liver, while the majority of LCA is excreted in feces.^114^ Besides, BAs as signal molecules and metabolic integrators stimulate nuclear FXR and membrane TGR5, and control cholesterol, lipid, and energy metabolism.^115^

#### FXR regulates BAs synthesis and transport in the gut-liver axis

**Fig. 5:**
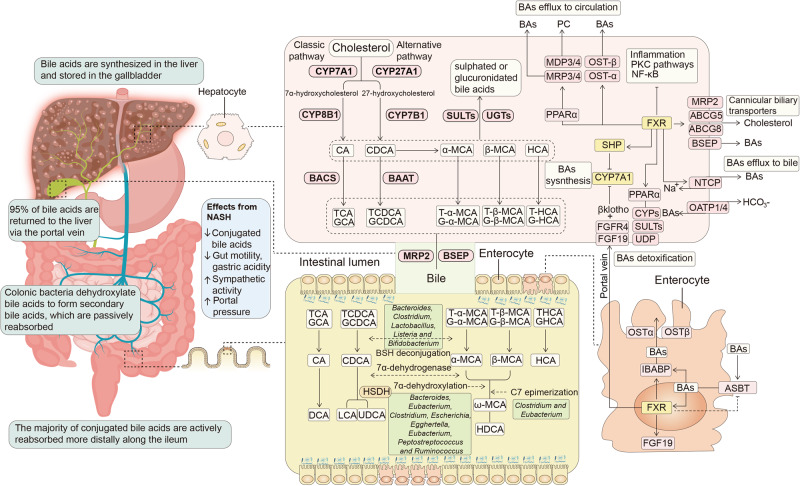
BAs biosynthesis, transport, and FXR-mediated BAs signaling in the gut-liver axis. BAs are synthesized in hepatocytes via CYPs-mediated oxidation of cholesterol to form CA and CDCA, which conjugate taurine, or glycine to form conjugated BAs and are secreted from the liver to the gallbladder and then into the intestine. Subsequently, gut microbes convert the primary BAs into secondary BAs. During gut dysbiosis, the expression of intestinal FXR is downregulated, leads to the increase of BAs synthesis and BAs influx, and the decrease of BAs efflux, and thus promote the progress of liver diseases. FXR also controls BAs detoxification and inflammation formation. Created with BioRender.com

FXR, which is highly expressed in the liver and the intestine tissues, is involved in the BAs metabolism in gut-liver axis (Fig. 5)^116,117^ and regulates a variety of critical metabolic pathways to maintain BAs homeostasis.^118^

In the intestine, apical sodium-dependent BA transporter (ASBT) induces BAs influx into the gut and promotes the ileal expression of FXR. In the meantime, FXR negative feedback suppresses the expression of ASBT,^119^ maintains homeostasis in the intestinal mucosa,^120^ boosts the expression of intestinal bile acid transport protein like organic solute transporter alpha/beta (OSTα/β), and induces BAs efflux.^121^ In addition, FXR promotes fibroblast growth factor 19 (FGF19) to enter the liver, and then suppresses CYP7A1 enzyme activity.

In the liver, FXR decreases CYP7A1 enzyme activity via FGF19-fibroblast growth factor receptor 4 (FGFR4) and FXR-small heterodimer partner (SHP) signaling pathways, and hence suppresses BAs production.^122^ FXR also indirectly down-regulates the expression of organic anion transporter 1/4 and BAs influx,^123^ and increases BAs efflux based on the up-regulation of OSTα/β, multidrug resistance-associated protein 3/4, BSEP and sodium-dependent taurocholate cotransporting polypeptide transporters.^124–127^

In addition, hepatic FXR up-regulates the transporters of choline and cholesterol like ABCG5/8 and MDP3/4^128,129^ and inhibits the expression of NF-κB and protein kinase C, which regulates the inflammation formation.^130^ FXR also increases the expression of peroxisome proliferator-activated receptor α and regulates BAs detoxification by encoding CYPs, sulfotransferases (SULTs) and UDP-glucuronosyltransferases (UGTs).^131^ All in all, FXR inhibits cholestasis and inflammation, and therefore suppresses the development of liver diseases.

#### TGR5 regulates BAs transport in the gut-liver axis

**Fig. 6:**
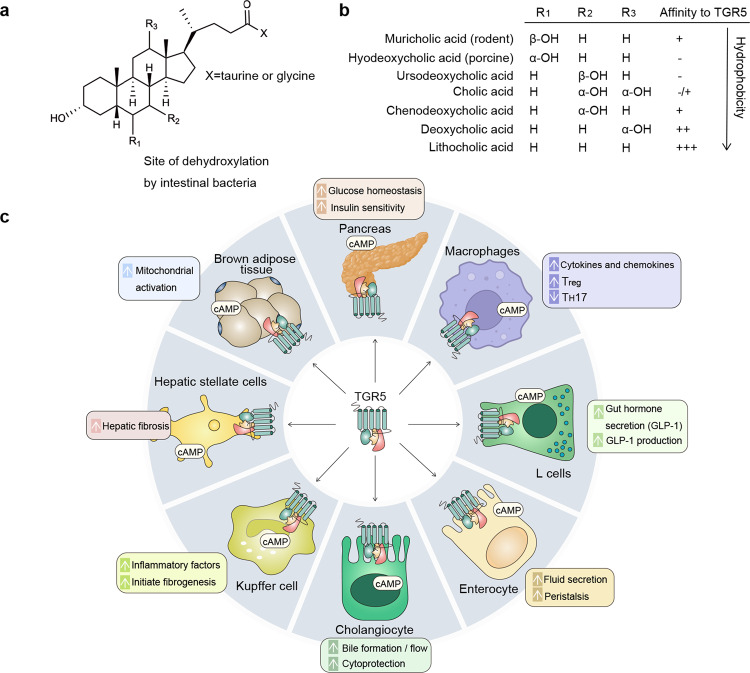
BAs as the messengers in the gut-liver axis activate TGR5. **a** The basic chemical structure of BAs. **b** BAs pool contains MCA, HCA, UDCA, CA, CDCA, DCA, and LCA. The OH group at R1, R2, or R3 and its spatial orientation determine the type of BA. R2 is the site of dehydrogenation. X is the site of conjugation. – to +++ represents the affinity from low to high based on their affinity to TGR5. **c** TGR5 is expressed in various cell types and tissues such as brown adipocytes, hepatic stellate cells, macrophages, pancreas, Kupffer cells, cholangiocytes, enterocytes, and L cells. Created with BioRender.com

TGR5 belongs to the transmembrane G protein-coupled receptor that is activated by BAs, increases the intracellular concentration of cyclic AMP (cAMP) and regulates BAs transport. LCA is the most potent natural TGR5 agonist among the BAs pool which contains muricholic acid (MCA), hyocholic acid (HCA), ursodeoxycholic acid (UDCA), CA, CDCA, DCA, LCA, and so on (Fig. 6a, b). TGR5 is found in a variety of cell types and organs such as brown adipocytes, hepatic stellate cells, macrophages, pancreas, Kupffer cells, cholangiocytes, enterocytes, and L cells (Fig. 6c).

On L cells, β cells and enterocytes, activation of TGR5 leads to the secretion of GLP-1,^132^ insulin^133^ and intestine peristalsis,^134^ which improves pancreas function and insulin sensitivity. On macrophages and Kupffer cells, activation of TGR5 dampens NF-κB-mediated cytokine expression, modulates immune signals of T_reg_ and T_H_17, and regulates immunity and inflammation.^100,135^ TGR5 on brown adipose tissue activates cAMP-dependent iodothyronine deiodinase 2 which converts inactive thyroxine into active thyroid hormone and regulates energy homeostasis.^136^ TGR5 on hepatic stellate cells promotes the formation of liver fibrosis.^137^ Besides, TGR5 on cholangiocytes regulates resorptive and secretory mediators, and modulates bile flow and composition^138^ (Fig. 6c).

#### Other BAs receptors regulate BAs synthesis and transport in the gut-liver axis

Shingosine-1-phosphate receptor 2 (S1PR2) as another G protein-coupled receptor exists in multiple hepatic cells, bile duct cells, hepatic stellate cells, intestinal endothelial cells and macrophages,^139^ binds to sphingosine 1-phosphate, and plays a differential role in multiple tissues.^140^ Importantly, BAs only in their conjugated form such as taurine or glycine conjugated BAs activate S1PR2.

S1PR2 existing around the liver and bile duct promotes hepatic fibrogenesis via influencing the activity of bone marrow-derived macrophages. S1PR2 deficiency dramatically decreases bile duct ligation-induced bile duct cell proliferation and bile stasis damage, as evidenced by a significant decrease in inflammation and hepatic fibrosis in S1PR2 knockout mice. Meanwhile, S1PR2 antagonist JTE-013 drastically lowers blood total BAs and cholestatic liver injury in mice with bile duct ligation.^141^

Besides, S1PR2 in intestinal endothelial cells is a key protein in maintaining intestinal mucosal barrier function. Inhibition of S1PRs2 restores gut barrier function and M1 macrophage polarization, and decreases ER stress of gut endothelial cells and glycolysis in macrophages.^142^

The vitamin D receptor (VDR) as the superfamily of nuclear receptors is implicated in immunity, cellular development, insulin production, and secondary bile acid detoxification. VDR has an affinity for dehydro-LCA and LCA,^143^ which is more sensitive than other BAs receptors. When VDR is activated, it stimulates the expression of cytochrome P450 3 A, which encodes cytochrome P450 enzymes responsible for LCA detoxification in the liver and gut.^144^ Meanwhile, the levels of VDR is linked to beta-diversity of gut microbiota which corresponds with enhanced Janus kinase (JAK)/ signal transducer of activators of transcription (STAT) signaling, as well as increased secondary BAs and intestinal tumor burden.^145^ In addition, the amount of *Lactobacillus* is decreased, while *Clostridium* and *Bacteroidetes* species are elevated which link to VDR-induced the change of BAs and fatty acids in VDR-deficient mice.^146^

Pregnane X receptor (PXR) as a well-known orphan nuclear receptor is enriched in the liver and intestine and responds to xenobiotic and BAs exposure.^147,148^ PXR is regulated by various endogenous substances, especially for microbial metabolites such as some secondary BAs and 3-indolepropionic acid.^149^ Among these metabolites, PXR has a better affinity for LCA than for DCA and CA.^149,150^ When PXR is lack, the relative abundance of *Lactobacillus* increases, which possesses bile salt hydrolase, and therefore hydrolyzes primary taurine-BAs in feces. Besides, PXR-deficient mice have a characteristic leaky gut physiology which is accompanied by an increase of the TLR signaling pathway.^151^

Retinoic acid-related orphan receptor (ROR γt) as a nuclear receptor is linked to a number of inflammatory and autoimmune disorders. Th17 cells,^152^ lymphoid tissue inducer cells,^153^ type 3 innate lymphoid cells,^154^ and T cells^155^ can express ROR γt receptor. Several inverse agonists containing cholesterol intermediates and oxysterols can lower ROR γt. These inverse agonists decrease the transcription binding activity of ROR γt and reduce the production of pro-inflammatory cytokines in inflammation and autoimmune disorders.^156^

### Affecting immunity in the gut-liver axis

The intestinal innate immune system plays an important role in providing the first line of defense against intestinal pathogens. The liver is a central immunological organ with a high exposure to circulating pathogens and endotoxin from the gut microbiota. It’s particularly enriched in multiple immune cells including macrophages, lymphoid cells, mucosal-associated invariant T (MAIT) and γδ T cells.

Intestinal macrophages release inflammatory signals and promote the hepatic recruitment of blood monocytes, which locally develop into monocyte-derived macrophages and increase the size of the macrophage pool in livers.^157^ Natural killer (NK) cells are a major population of lymphocytes in the liver. They release immunomodulatory cytokines including IFNγ, IL-4, and IL-13 to damage the intestinal barrier. Liver-resident NK cells have many similar characteristics to immune-regulatory lymphocytes (known as innate lymphoid cells), which are frequently present on the intestinal mucosal surfaces of the gut.^158^ In addition, MAIT cells take part in multiple liver pathogenesis, and inhibit liver inflammation and damage.^159,160^ γδ T cells as another type of innate-like T cells exist in the steady-state liver, whose development is sustained in a microbiota-dependent way. The increase of γδ T cells in the liver causes hepatic damage.^161^

## Mechanisms linking the gut-brain axis

**Fig. 7:**
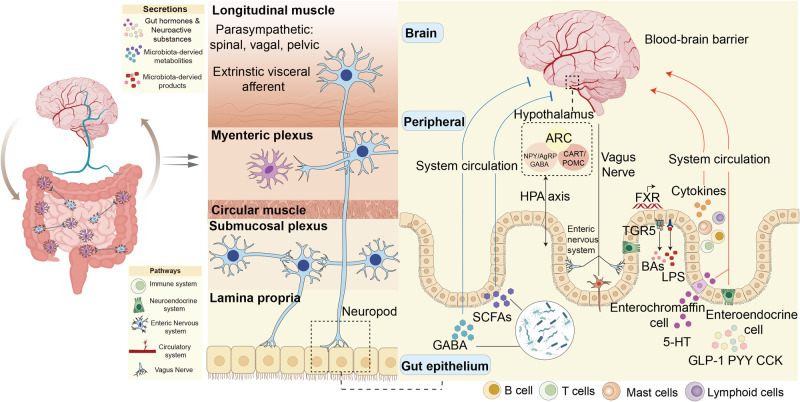
The mechanisms linking the gut-brain axis. Gut microbiota is capable of synthesizing neurotransmitters like SCFAs and GABA, which have different peripheral and central effects on modifying host metabolism and central regulation of appetite directly via vagal stimulation or indirectly through immune-neuroendocrine mechanisms. Enteroendocrine cells are activated by these microbial-derived metabolites, and lead to the production of gut hormones such as 5-hydroxytryptamine (5-HT), GLP-1, PYY, and cholecystokinin (CCK). These gut hormones are released from the gut to the nucleus tractus solitarius of the brain via the vagus nerve and direct secreted into the circulatory system. Information from the nucleus tractus solitarius is distributed to the arcuate nucleus (ARC) in the hypothalamus, where appetite and energy balance are regulated. The ARC contains neuropeptide Y, agouti-related protein, anorexigenic peptides, cocaine amphetamine-regulated transcript, and pro-opiomelanocortin neurons. Moreover, gut microorganisms also use bile acids and their conjugates to activate FXR and TGR5, and increase GLP-1 secretion by enteroendocrine cells. Additionally, gut microbiota is associated with inflammation via the release of LPS, which activates immune cells, such as B cells and dendritic cells, and promotes the production of cytokines. Created with BioRender.com

**Fig. 8:**
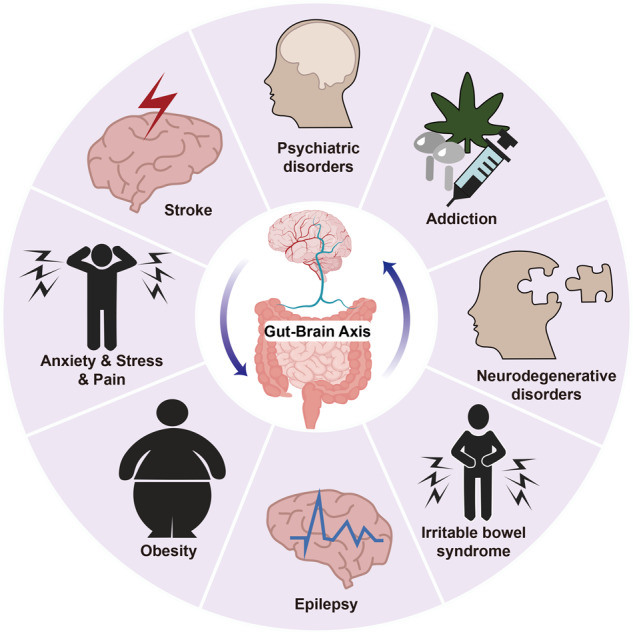
An outline map of various diseases in the gut-brain axis is currently implicated including psychiatric and neurodegenerative disorders, pain, stress, IBS, stroke, addiction, and obesity. Created with BioRender.com

Gut-brain axis is mediated based on the circulatory system, vagus nerve,^162^ immune system,^163^ neuroendocrine system,^164^ and ENS.^165^ There is a wide range of neuroactive substances, including gut hormones, neuroactive compounds, gut microbiota-derived metabolites, and gut microbiota-derived products in this axis (Fig. 7).^166^ After metabolites enter the brain, they affect neurological growth and neuronal degeneration under many situations including social and cognitive behavior, fear, stress, and food intake. Furthermore, the brain feeds back to the gut and paracrine through the vagus nerve (Fig. 8).^167,168^

### Changing BBB in the gut-brain axis

The BBB is a barrier that prevents diffusion between the circulatory system and central nervous system (CNS) cerebrospinal fluid.^169^ The gut microbiota and its metabolites regulate the expression of TJs proteins, lead to the release of inflammatory cytokines,^170^ and further induce the structural change of the BBB.^171,172^ Under normal physiological conditions, cytokines are difficult to pass through the BBB and affect brain regions.^173^ Once gut disorders occur, the large amounts of IL-1 and IL-6 change BBB permeability, pass through BBB, activate the hypothalamic-pituitary-adrenal (HPA) axis and produce cortisol, which is the most potent stimulator for the pressure system.^174^ Meanwhile, psychological or physical stress significantly disrupts the HPA axis, which mainly regulates stress response and has a significant impact on the gut-brain axis,^175^ especially in IBS.

### Changing vagus nerve in the gut-brain axis

The vagus nerve serves as a significant two-way highway that connects the brain to the gut.^176^ It is intrinsically linked to ENS, which alters brain behavior like stress reactivity, anxiety, depressive, and social behaviors as well as cognition.^177–179^ The afferent fibers of the vagus nerve come from the intestinal smooth muscle, and transmit information from the intestine to the CNS.^180,181^ Furthermore, the vagus nerve may detect microbial messages from CNS and then feed back to the intestine, release bacteria-derived metabolites such as SCFAs, GABA, and 5-HT, or gut hormones like GLP-1, PYY, and CCK, which are affected by enteroendocrine and enterochromaffin cells in the intestinal epithelium.^26,182,183^

### Regulating neurotransmitters in the gut-brain axis

The gut microbiota can synthesize and modulate their hosts to produce neurotransmitters^184^ including GABA, glutamate, acetylcholine, dopamine, norepinephrine and trace amines.^13^

#### GABA in the gut-brain axis

GABA as gut bacteria-derived metabolites is an amino acid derivative of glutamate, which is widely distributed in the mammalian CNS and significantly modulates synaptic suppression and effects on psychological diseases including behavioral disorders, insomnia, and pain.^185,186^ GABA is produced by several bacteria, including *Bacteroides, Bifidobacterium, Lactobacillus and Escherichia spp*, which involves ENS homeostasis and disturbance, such as acid secretion, gastric emptying, bowel motion, and sensation of pain.^187,188^

Importantly, the adhesion from GABA to GABA receptors, and then to postsynaptic neurons inhibits the transfer of Na^+^, K^+^, Ca^2+^, and Cl^−^.^189^ Three classes of GABA receptors include GABA-A, GABA-B, and GABA-C, which transfer signals received from hormones, neurotransmitters, and pheromones. Among them, GABA-A^190^ and GABA-C^191^ receptors belong to ionic receptors, while GABA-B^192^ receptors are metabolic receptors. GABA-activating GABA receptors exert a depressant effect and alter behavior in the mammalian CNS.^187^ GABA-A receptors are the primary inhibitory neurotransmission receptors in the CNS, which means they are involved in the majority of brain physiological processes.^193^ Besides, gut nerve cells releasing GABA activate GABA-A receptors in the ENS instead of suppressing neurons in the CNS and raise the levels of intracellular chloride via sodium-potassium-chloride transporters.^194^ Oral administration of gut bacteria like *Lactobacillus spp* increases blood expression of GABA and the number of GABA-A receptors in the brain, which benefits intestinal health, stress-like behaviors, and growth performance.^195,196^

#### Glutamate in the gut-brain axis

Glutamate as the metabolic precursor of GABA is the most abundant excitatory neurotransmitter in the brain. Gut microbiota such as *Bacteroides vulgatus Lactobacillus plantarum, Lactobacillus paracasei, Lactococcus lactis* and *Campylobacter jejuni* produce glutamate, and improve cognitive functions and behavior.^197^ Glutamate regulates the gut-brain axis based on CNS and vagus nerve.^198^ Glutamate from daily diet or gut microbiota cannot cross the BBB in CNS. Its production in brains is dependent on the collaboration of neurons and astrocytes using intermediate metabolites of glycolysis, and phosphate-activated glutaminase from hydrolytic deamination of glutamine.^199^ Besides, a subgroup of intestinal enteroendocrine cells can also synthesize glutamate and transmit its downstream signals fast to the brain through the vagus nerve. Neuropod cells belonging to enteroendocrine cells synapse with the vagus nerve, boost expression of vesicular glutamate transporter 1 and produce glutamate to send sensory information from sugars in the gut to the brain in milliseconds, which may improve multiple brain diseases.^200,201^

#### Acetylcholine in the gut-brain axis

Acetylcholine as a gut bacteria-derived product is a common cholinergic neurotransmitter, which plays a local mediator in the central and peripheral nervous system by transmitting excitation signals between neurons.^202^ For example, increased acetylcholine relieves symptoms in AD,^203^ increases expression of TJs in the colon and hippocampal tissue,^204^ and prevents cognitive impairment. A variety of gut bacteria, such as *Bacillus subtilis*, *Escherichia coli, Lactobacillus plantarum*, and *Staphylococcus aureus* secrete acetylcholine.^205^ Among them, *Bacillus subtilis* releases more acetylcholine than *Escherichia coli* and *Staphylococcus aureus* does.^206^ Generally, acetylcholine cannot cross the BBB. Neurons in the CNS catalyze choline and acetyl-CoA to synthesize acetylcholine based on choline acetyltransferase. Meanwhile, peripherally derived choline crosses the barrier to reach the brain.^207^

#### Dopamine in the gut-brain axis

Dopamine as a gut bacteria-derived product is a kind of catecholamine neurotransmitter in the brain.^208^ It regulates various physiological functions in CNS-related diseases^209^ such as PD and schizophrenia. Dopamine and its receptors are found throughout the gut, where they influence intestinal functions like mucosal blood flow, gastric secretion and motility.^210,211^ Dopamine is rich in diet and can be transported to the brain through the BBB.^212^ Besides, the gut microbiota like *Staphylococcus* produces more than half of the dopamine in the body. They can absorb and convert the precursor l-3,4-dihydroxy-3phenylalanine into dopamine depending on the staphylococcal aromatic amino acid decarboxylase.

#### Norepinephrine in the gut-brain axis

Norepinephrine as a gut bacteria-derived product is another kind of catecholamine. Although its content is small, it acts as a neurotransmitter in the central and peripheral nervous system.^213^ It involves behavior and cognition such as memory, learning, attention, arousal, and alertness. It also triggers acute stress reactions in threatening situations. Norepinephrine is mainly synthesized and secreted by the adrenal medulla. In the brain, norepinephrine is produced by locus coeruleus neurons where the precursor of the neurotransmitter tyrosine is converted into dopamine, and finally forms norepinephrine. Importantly, changes in intestinal microbiota composition at low temperature can regulate the release of norepinephrine from the gut and brown adipose tissue of *Lasiopodomys brandtii* via the cAMP signaling pathway, thus helping to regulate energetics and thermogenesis.^214^

#### Trace amines in the gut-brain axis

Trace amines as gut bacteria-derived products contain *β*-phenylacetylene amine, *p*-pyrimidine, tryptamine, *p*-octopamine, and so forth.^215^ Although their abundance in the brain is very low, they are regarded as important nerve modulators or neurotransmitters. In common, trace amines are rich in ordinary food and can be produced and degraded by gut microbial.^216^ For example, *Staphylococcus* strains in the gut express staphylococcal aromatic amino acid decarboxylase, decarboxylate its corresponding aromatic amino acid substrates and synthesize three types of trace amines including tryptamine, tyramine, and phenethylamine through the decarboxylation of its corresponding aromatic amino acid substrates.^216^ Besides, *Clostridium sporogenes* and *Ruminococcus gnavus* in the gut decarboxylate tryptophan with their own tryptophan decarboxylase and produce tryptamine.^217^

### Changing gut hormones and other microbial metabolites in the gut-brain axis

#### Gut hormones in the gut-brain axis

Microbiota-mediated enteroendocrine and enterochromaffin cells in the intestinal epithelium can release multiple gut hormones, including 5-HT, PYY, GLP-1, CCK, and ghrelin, which regulate multiple brain disorders, such as anxiety and depression.^218,219^

5-HT represents one of the most distinctive gut hormones and is generated by enterochromaffin cells. It possesses extensive receptor subtypes and gastrointestinal tract locations,^220^ including the stomach, small intestine, and large intestine, which regulates intestinal motility and perception of pain in the peripheral nervous system and modulates emotions, sleep, and appetite in the CNS.^218^ Despite peripheral and central 5-HT are generated differently and divided by the BBB, they are deeply related to CNS.^221^ First, tryptophan as a 5-HT precursor has multiple benefits for CNS and ENS functioning in the gut-brain axis. The metabolism of tryptophan based on the kynurenine pathway in the peripheral tissues has a significant impact on CNS.^222,223^ Second, 5-HT plays a crucial role in innate as well as adaptive immunity.^224^ A recent investigation discovers that endotoxin injection promotes the release of 5-HT from platelets into the plasma and further stimulates lymphocytes and monocytes to secrete cytokines and regulate CNS functioning.^224,225^ Third, 5-HT produced from enterochromaffin cells also alters vagal afferent action, which in turn changes the gut-brain axis. A recent study displays that chemotherapy causes a quick release of 5-HT release, and thereby induces nausea and emesis, which mainly depends on the stimulation of vagal afferents in the intestines.^226,227^

Stress-related diseases, neural protection, neurological inflammation, and neurogenesis are all affected by the PYY, which may activate Y4 receptors and then engage in anxiety and depression regulation.^228^ GLP-1 is well-recognized as a hormone that stimulates glucose-dependent insulin production, and also responds to brain diseases like PD and depression via the GLP-1 receptor. Importantly, endogenous or exogenous glucocorticoids decrease GLP-1 bioavailability.^229,230^

CCK is widely generated in the CNS and peripheral nervous system and is primarily involved in the regulation of calorie intake^219^ and anxiety-related actions.^231,232^ CCK affects neurotransmitters including glutamate, dopamine, acetylcholine, and GABA, which have an impact on the function of the brain.

Ghrelin, recognized for its adipogenic and orexigenic function, is discovered as a stress response, anxiety, and depression regulator.^233,234^ Numerous stressors such as restraint stress and social defeat raise the levels of ghrelin. Importantly, after hunger, the levels of ghrelin increase and cause stress adaption.^235^ Ghrelin receptor agonists enhance fear memory generated by stress while its antagonists decrease fear memory, demonstrating that ghrelin increases anxiety and depression-like behaviors.^236^

#### SCFAs in the gut-brain axis

SCFAs as gut bacteria-derived metabolites are transported from the gut to the CNS, cross the BBB with the bloodstream,^237^ and act as signals to affect host metabolism and immunity reaction, which has a substantial impact on human physical and mental health.^238,239^ For example, SCFAs act on homologous free fatty acid receptors or taste receptors, and regulate intestinal physiological functions including movement, secretion, and inflammation.^240,241^ Meanwhile, SCFAs pass through the BBB, enter the CNS, reduce LPS-induced neurological inflammation in primary microglia and hippocampus, and decrease circulating pro-inflammatory cytokines.^242,243^ Therefore, SCFAs are linked to a variety of disorders including anorexia, inflammatory bowel disease, neurological inflammation, and so forth.^244,245^

#### Other microbial metabolites in the gut-brain axis

Other microbial metabolites such as BAs and TMAO directly connect with the nervous system to maintain body growth and development.^246,247^

BAs are synthesized in hepatocytes, secreted into the intestine and metabolized by gut microbiota. They significant impact on the body, particularly on the brain.^248,249^ For example, gut dysbiosis causes secondary BAs shortage in patients with IBS and intensifies the pro-inflammatory mediators in the CNS, which can be reversed through the increase of secondary BAs activated by TGR5. This pathway is regulated by the increase of secondary BAs activated by TGR5.

Secondary BAs also stimulate FXR transcription in the ileum, and further trigger the synthesis of FGF19, which has the ability to move into the bloodstream, crosse the BBB, and trigger the hypothalamic ARC.^250^ Subsequently, the hypothalamus regulates glucose homeostasis and inhibits HPA function.^251^ By boosting GLP-1 secretion from L cells through TGR5 signaling, it has a vital function in managing glucose metabolism, and thereby influences the uptake behavior and food intake.^23^ In addition, restoring the gut BAs pool in mice with malnutrition increases the amount of gut intraepithelial lymphocytes like RORγt^+^ Treg cells, reduces the host’s sensitivity to colitis via BAs nuclear receptors, and lowers the risk of neurological inflammation.^155^ Additionally, gut dysbiosis causes secondary BAs shortage in patients with IBS and intensifies the pro-inflammatory mediators in the CNS, which can be reversed by TGR5, which increases secondary BAs.^252^

Besides BAs, TMAO is mostly produced by the gut microbiota through the metabolism of choline and betaine, and exerts a direct influence on the CNS.^252^ For example, TMAO accelerates brain aging and causes age-associated cognitive impairments.^253^ Moreover, microbiota-related TMAO directly communicates with the mammalian BBB, with implications for cerebrovascular and neurological health.^254^

### Affecting immunity in the gut-brain axis

#### Innate immunity in the gut-brain axis

The nervous system and innate immune system have capacity to quickly identify and react to potentially harmful signals like TLRs for pathogen detection and other damage-associated molecules.^255^ Neuron interacts with three gut-resident innate immune cells, including macrophages, lymphoid cells and mast cells.^256^

Macrophages are present throughout the entirety of the gastrointestinal tract and are crucial for innate immunity. They connect with smooth muscle, capillary cells and glial cells, devour pathogens, absorb microbial products, and maintain ENS homeostasis.^257,258^ The unique population of macrophages known as muscularis macrophages regulate gut motility,^259^ release macrophage growth factors like colony-stimulating factor 1 (CSF1),^260^ and secrete bone morphogenetic protein 2 (BMP2) to alter gut peristalsis activity.^261^ Gut microbiota regulates the expression of BMP2 and CSF1 in intestinal nerves. Therefore, there is an easily modifiable microbiota-driven interaction between macrophages and intestinal nerves that regulate gastrointestinal motility.^260^

Although lymphoid cells share the same lymphoid progenitor with lymphocytes, they are regarded as barrier resident lymphocytes and belong to the innate immune system, which initially responds to tissue injury. As the innate counterparts of T cells, lymphoid cells do not have T cell receptors generated by antigen-specific receptor somatic cell recombination.^262^ However, they effectively regulate the host’s defense and immunological reaction.^257,263^ For the maintenance of gut homeostasis and inhibiting pathogen infection, neurons positively interact with both type II and type III lymphoid cells.^264^ Besides, they take an important part in the early stages of the immune reaction by swiftly reacting signals or cytokines generated by other cell types.^265^

Mast cells exist in the mucosal and submucosal layers of the intestine and have a tight anatomical relationship with sensory and autonomic nerve terminals.^266,267^ There are numerous receptors binding to typical neurotransmitters such as acetylcholine, corticotropin-releasing hormone, and neuropeptides including substance P, calcitonin gene-related peptides, and hemokinin in mast cells. The function of mast cells is influenced by those nerve-derived substances.^268^ For example, during stress, corticotropin releases hormone secretion, leads to hypercortisolism, promotes mast cell maturation, and induces neurogenic inflammation.^269^

#### Adaptive immunity in the gut-brain axis

Adaptive immunity plays an important roles in modulating interactions between the intestine and brain.^270^ LPS as an endotoxin exists in the cellular wall of gram-negative microbes that generates endotoxaemia, elicits an extensive immunological response and activates adaptive immune cells,^271^ such as B and T cells, which can serve as sensors of bacterial, present within the gut, convey signals to the enteric neural system and result in alterations to ENS.^272^

CD4^+^ and CD8^+^ T cells as core regulators of adaptive immunity interact with the peripheral nervous system.^273^ In the cholinergic anti-inflammatory reflex, the efferent nerve of the vagus nerve delivers the message to the abdominal ganglia, subsequently to the spleen through β2 adrenergic receptor and later conveys them to choline acetyltransferase^+^ T cells that generate acetylcholine.^274^ T cell-released acetylcholine acts on nicotinic acetylcholine receptors in macrophages and prevents the release of TNF.^275^

When B cells are activated, they transform from IgM-producing plasma cells to IgA-producing plasma cells, which increase the reaction of B cells to microorganisms and pathogens.^276^ It’s still uncertain whether particular neurons connect directly or indirectly with B cells in the gut. In mice with autoimmune encephalomyelitis, colonic motility is decreased, while glial fibrillary acid protein expression is increased and accompanied by increased immunoreactivity towards ENS neurons and glial cells likely due to B cell immunoglobulin synthesis increased.^275^

## Mechanisms linking the liver-brain axis

Inter-organ communication between the liver and brain occurs via the signaling between the nervous and circulatory system.^277^ The mechanism of liver-brain axis mainly includes BBB permeability, vagus nerve, epigenetic regulation, toxic metabolites, β-amyloid (Aβ) metabolism, and immune response.

### Changing BBB in the liver-brain axis

Changing the permeability of BBB by proinflammatory cytokines such as TNF and IL-1β in the liver causes the indiscriminate entry of toxins such as ammonia and xenobiotics, which produce a proinflammatory response.^278^ For example, BBB is destroyed in mice with acute liver failure, causes TNF and IL-1β to cross the BBB, and further impairs brain function.^279^

### Changing vagus nerve in the liver-brain axis

The hepatic vagal sensory afferent nerves are responsible for indirectly sensing the liver microenvironment and relaying the sensory inputs to the nucleus tractus solitarius of CNS, and then feeding back to the liver vagal parasympathetic nerves.^280,281^ For example, the signals from the brain regulate VLDL triglyceride secretion and reduce hepatic lipid content via the vagus nerve.^282^ Besides, an exogenous vagal reflex activity connects hepatic vagal sensory inputs, brainstem, vagal efferents, and intestinal neurons.^281^

### Epigenetic regulation in the liver-brain axis

Methylation is important for development, imprinting, transcriptional control, chromatin structure, and overall genomic stability.^283^ Hepatic DNA, RNA, and histone methylation are most likely involved in brain development.^284^ Preeclampsia causes changes in the DNA methylation of numerous critical regulatory genes in the fetal brain and liver, which indicates that liver-brain axis exists.^285^ Besides, one typical posttranscriptional regulator of mRNA is RNA N6methyladenosine, which is associated with brain activities.^286^ β-hydroxybutyrate is produced by the liver and goes through the bloodstream to the brain, where it inhibits histone deacetylases.^287^ Furthermore, injecting β-hydroxybutyrate to the brain results in an increase in brain-derived neurotrophic factor, which is used to treat mental conditions like depression and neurodegenerative disorders.^288^

MicroRNAs are a kind of tiny, tissue-specific, non-protein-coding RNA that maintains cellular homeostasis by regulation of negative genes in the liver-brain axis. MicroRNAs regulate hepatic lipogenesis and critical brain functions.^289^ Dysregulation of microRNAs is linked to a variety of liver and cerebral diseases. For example, miR212/132 is expressed in the brain and is sensitive to external cues from the liver. Brain-derived exosomes, known as transport microRNAs, are detected in the bloodstream and liver.^290^

### Toxic metabolites in the liver-brain axis

#### Ammonia

Ammonia is involved in the pathophysiology of liver-brain axis.^291^ Astrocytes absorb hepatmogenic ammonia and convert it into glutamine. Glutamine buildup in astrocytes exerts an osmotic impact, causes cerebral edema and neuronal cell death via *N*-methyl-d-aspartate receptor overactivity, and leads to the formation of Alzheimer’s type 2 astrocytes, which have a bloated appearance and larger nuclei.^292^ Ex vivo feeding of ammonium salts to healthy animals causes microglial activation as well as elevates expression levels of IL-1β in the brain.^293^ There is also a strong relationship between circulating ammonia and the levels of TNF in individuals caused by chronic liver failure.^292^ For example, proinflammatory gene like TNF-α up-regulates in the brains of cirrhotic hepatic encephalopathy (HE) individuals.^294^ Meanwhile, TNF-α exposing to human cerebrovascular endothelial cells increases ammonia absorption.^295^

#### Lactate

The brain concentration of lactate is increased in the acute and chronic liver failure models,^296^ which correlates with the severity of clinical symptoms, electroencephalogram spectral abnormalities, and degree of microglial activation.^297^ At the coma phases of encephalopathy in liver failure, brain concentration of lactate reaches 10–12 mM,^298^ which triggers high concentration of TNF and IL-6 released from microglial cell. Lactate accumulation in the brain is also linked to the ammonia-inhibited ketoglutarate dehydrogenase and phosphofructokinase 1.^299^

#### Manganese

Manganese deposition in the basal ganglia region of the brain usually occurs in cirrhotic individuals,^300^ because of the poor hepatobiliary metal removal and portal-systemic shunting. Manganese deposition is also associated with bilateral T1-weighted signal hyperintensities on magnetic resonance imaging (MRI) as well as dopaminergic cell death in these tissues, providing a compelling explanation for the high occurrence of parkinsonism in cirrhosis. There is substantial evidence that neuroinflammatory processes are involved in the neurotoxic effects of manganese.^301^ Manganese, for example, is proposed to regulate inflammatory cytokine output from microglia as well as to induce microglia to emit hydrogen peroxide and nitric oxide.^302^

### Aβ metabolism in the liver-brain axis

Imbalanced Aβ generation and clearance are hypothesized to play an important roles in the development of AD.^303^ The Liver is the main site for peripheral Aβ metabolism whose disorder may lead to AD progression.^304^ These disturbances are further exacerbated by the pro-inflammatory condition that frequently accompanies liver illnesses, resulting in neuroinflammation.^305^ Meanwhile, the present eating habits like the Western diet change the bile acid profile in the liver, and also link to both AD and PD. Supplementation with Aβ ameliorates these diseases.^306^

Besides, Aβ metabolic disorders cause oxidative stress and inflammation, which further lead to chronic hepatic and neural diseases.^305^ For example, hepatic oxidative inflammation is characterized by the dysregulation of antioxidant enzymes and the HPA axis, as well as the release of pro-inflammatory cytokines such as IL-6 and TNF-α. The oxidative stress factors in the liver are linked to neophobia. These changes might pave the way for a novel route and the identification of prospective integrative system targets for liver-brain axis research.^307^

### Immunity in the liver-brain axis

Some immune cells such as macrophages, dendritic cells and lymphocytes release proinflammatory factors like TNF-α and IL-1β. These inflammatory factors further promote the release of secondary messengers such as prostaglandins and nitric oxide from cerebral endothelial cells, and cause alterations inside the brain.^308^ For example, the proinflammatory cytokines such as TNF-α and IL-1β induce the release of the inducible nitric oxide synthase (NOS) isoform from macrophages and cerebral endothelial cells.^309^ In this process, nitric oxide is produced through the NOS-oxidized L-arginine in endothelial and neuronal cells.^310^ In addition, inhibition of NOS promotes anxiolytic effects in rats.^311^

Microglial activation usually attracts monocytes into the brain, and causes chronic inflammatory diseases in patients with liver failure.^312,313^ For example, microglia releases TNF and triggers monocyte recruitment in livers. Microglia also secretes chemokine (CC-motif) ligand 2, facilitates liver monocyte migration into the brain,^314^ and causes neurological problems in mice with biliary cirrhosis. These discoveries represent a new liver-brain communication route, which leads to increased neuronal excitability and neurological problems in liver disorders.

IL-6 as another proinflammatory factor influences brain function via liver-brain axis. The hepatocytes and leukocytes generate IL-6 and express the IL-6 receptor (IL-6R) in the cell surface when they are activated. The IL-6/IL-6R complex subsequently interacts with the transmembrane glycoprotein which exists on the cerebral endothelial cells, and initiates the signaling cascade.^315,316^ For example, bile duct ligation induces hepatic inflammation and sickness behaviors accompanying with the increased levels of hepatic IL-6 and circulatory IL-6. The sickness behaviors are significantly reduced in IL-6 deficient mice and increased by intravenous injection of recombinant IL-6.^317^

## Therapies targeting the gut-liver-brain axis

### Antibiotics application

Antibiotics, especially non-absorbable antibiotics, mainly stay in the intestine, regulate the intestinal microbiota and affect the gut liver brain axis disease progression.^318^ For example, rifaximin as a non-absorbable, broad-spectrum and gastrointestinal-specific antibiotic display effective and safe in biopsy-proven NAFLD.^319^ It decreases serum levels of endotoxin, proinflammatory cytokines and cytokeratin (CK)-18, but has no effect on the hepatic lipid content, body mass index (NCT02884037), the serum levels of alanine aminotransferase (ALT), peripheral glucose uptake or hepatic insulin sensitivity (EudraCT 2010-021515-17).^320^ Meanwhile, rifaximin treatment significantly decreases the relative abundance of gut microbiota including *Peptostreptococcaceae, Verrucomicrobiaceae* and *Enterobacteriaceae*, but belongs to a minor, temporary effect on a wide variety of gut bacteria in a 2-week open-label IBS clinical trial (Table 3).^321^ Other multiple randomized controlled trials with rifaximin also indicate its minor therapeutic improvements in patients with IBS.^322^ The fact that gut microbial dysbiosis is not a causal factor in IBS symptoms. Several negative trials of fecal microbial transplantation in patients with IBS provide similar data.^323^ Therefore, further study should reveal if rifaximin impacts IBS.

Solithromycin as a potent next-generation macrolide antibiotic reduces NAFLD activity score (NAS) and the levels of ALT in a phase II 13-week open-label NASH trial (NCT02510599). It also promotes the proliferation of some common gut microbiota like *Bifidobacteria* and *Lactobacilli* in short-term treatment,^324^ but disturbs flora balance in long-term studies (Table 2).^325^ Additionally, some antibiotics like ampicillin and amoxicillin increase the risk of endocarditis^326^ and bacteremia.^327^

**Table 2:** Clinical trials targeting gut-liver axis

Classification	Formula	Study status	Disease	Phases	NCT number
Antibiotics	Solithromycin	Completed	NASH	II	NCT02510599
	Rifaximin	Positive^438^	HE	III	NCT02016196
		Positive^438^	HE	IV	NCT02019784
Probiotic	*Streptococcus thermophilus, Bifidobacterium* and *Lactobacillus*	Terminated	Fatty Liver	I	NCT00099723
		Positive^330^	Obesity	NA	NCT01650025
	*Lactobacillus rhamnosus GG, Bifidobacterium breve BR03, Lactobacillus plantarum*	Not yet recruiting	NASH	NA	NCT04781933
	*Lactobacillus acidophilus, Lactobacillus casei subsp, Lactobacillus lactis, Bifidobacterium bifidum, Bifidobacterium infantis* and *Bifidobacterium longum*	Unknown	NAFLD	NA	NCT04074889
	*Bifidobacterium*	Terminated	NAFLD	I/II	NCT04175392
	*Lactobacillus acidophilus* and *Bifidobacterium lactis*	Unknown	NAFLD	NA	NCT02764047
	*Lactobacillus Lactococcus, Bifidobacterium, Propionibacterium* and *Acetobacter genera*	Completed	NAFLD	NA	NCT03528707
			NAFLD		NCT03614039
	*P. Pentosaceus, L. Lactis or L. Helveticus*	Unknown	NAFLD	NA	NCT04555434
	*Streptococcus thermophilus, Bifidobacterium* and *Lactobacillus*	Terminated	NASH	I/II	NCT03511365
	-	Positive^439^	NAFLD	NA	NCT00870012
	*Lactobacillus rhamnosus strain GG*	Recruiting	NAFLD	NA	NCT04671186
	*Lactoplantibacillus plantarum* and *Levilactobacillus brevis*	Completed	NAFLD	NA	NCT04823676
	*Lactobacillus reuteri GMNL-263* and *GMNL-89* and *Lactobacillus rhamnosus GMNL-74*	Recruiting	NAFLD	NA	NCT05402449
	*Lactobacillus acidophilus, Bifidobacterium lactis, Lactobacillus rhamnosus* and *Lactobacillus paracasei*	Unknown^440^	NASH	NA	NCT03467282
	*Bifidobacterium bifidum* and *Lactobacillus plantarum* 8PA3	Positive^441^	ALD	NA	NA
	*Lactobacillus casei Shirota*	Positive^442^	ALD	NA	NA
	*Lactobacillus subtilis* and *Streptococcus faecium*	Positive^443^	AH	NA	NA
	Inulin-type fructans	Positive^344^	Healthy	NA	NCT03042494
	Oligofructose	Positive^343^	NASH	NA	NCT03184376
	Inulin and oligofructose	Active not recruiting	NAFLD	NA	NCT02642172
	Prebiotic fiber	Positive^444^	Obesity	NA	NCT02125955
	Prebiotic fiber	Active not recruiting^445^	NAFLD	NA	NCT02568605
	Polyphenols	Positive^359^	NAFLD	II/III	NCT03380416
Synbiotics	Fructo-oligosaccharides & *Bifidobacterium*	Negative^446^	NAFLD	NA	NCT01680640
	Fructo-oligosaccharides & *Lactobacillus rham- nosus, Lactobacillus acidophilus, Streptococcus thermophilus, Bifidobacterium breve*	Positive^345^	NASH	II/III	NCT01791959
FXR	Obeticholic acid	Positive^380^	NASH	II	NCT01265498
		Positive^379^	NASH	III	NCT02548351
			NASH	III	EudraCT 20150-025601-6
	EDP-305	Completed	NASH	I	NCT03748628
		Completed^381^	NASH	II	NCT03421431
	MET409	Not recruiting	NASH	II	NCT04702490
	EYP001a	Completed	NASH	I	NCT03976687
		Recruiting	NASH	II	NCT03812029
	Nidufexor	Terminated	NASH	II	NCT02913105
	Tropifexor	Terminated	NASH	II	NCT02855164
	Cilofexor	Positive^382^	NASH	II	NCT02854605
	TERN-101	Positive^383^	NASH	I	NA
FGF19	Aldafermin	Positive^399,447^	NASH	II	NCT02443116
GLP-1	Liraglutide	Positive ^392,393,448^	NASH	II	NCT01237119
	ALT-801	Completed	NASH	I	NCT04561245
	Dulaglutide	Not recruiting	NASH	IV	NCT03648554
	Semaglutide	Negative ^395,396^	NASH	II	NCT02970942
FMT	A thin and healthy donor	Positive^449^	NAFLD	I/II	NCT02496390
	*Lachnospiraceae* and *Ruminococcaceae* donor	Positive ^450,451^	HE	I	NCT03152188
	A thin and healthy donor	Negative^449^	Obesity	I/II	NCT02496390
	A thin and healthy donor	Negative^452^	Obesity	I/II	NCT02530385
	A thin and healthy donor	Positive^453^	Obesity	I/II	NCT02741518
	A thin and healthy donor	Positive^454^	AH	NA	NA
Polyphenols	Polyphenols	Positive^455^	Obesity	NA	NCT02381145
					NCT01675401
PUFAs	Omega-3 fatty acids	Completed	NASH	II	NCT01056133
		Completed	NASH	II	NCT00845845
		Negative^388^	NASH	II/III	NCT00681408

Clinical study status is classified into seven categories: positive, negative, recruiting, not recruiting, completed, terminated, withdrawn, and unknown. Positive indicates the primary endpoint has been reached. Negative indicates that the primary endpoint was not met or that there were substantial adverse effects in the trial. The data is available in Clinical Trials (https://www.clinicaltrials.gov)

### Probiotics, prebiotics and synbiotics application

Probiotics are regarded as an adequate number of live microorganisms exerting beneficial effects on the host.^328^ The most commonly used probiotics in current studies contain *Lactobacilli*, *Streptococci*, and *Bifidobacteria*, which significantly decrease the development of liver and brain-related diseases^100^ such as NAFLD, NASH, ASD, depression, PD, schizophrenia, epilepsy, migraine, and so on.^329^ For example, VSL#3 as a probiotic mixture consists of eight distinct microbes such as *Bifidobacterium breve*, *Bifidobacterium infantis*, *Bifidobacterium longum*, *Lactobacillus acidophilus*, *Lactobacillus bulgaricus*, *Lactobacillus plantarum, Lactobacillus casei*, and *Streptococcus thermophilus*.^31^ It’s employed in NAFLD and obese children for four months. In the end, VSL#3 supplementation activates GLP-1, and alleviates fatty liver and body mass index (NCT01650025).^330^ Multistrain probiotics treatment including *Bifidobacterium bifidum*, *Bifidobacterium longum*, *Enterococcus faecalis*, *Enterococcus faecium*, *Lactobacillus acidophilus, Lactobacillus gasseri*, *Lactobacillus reuteri*, and *Lactobacillus rhamnosus* displays effective for the treatment of constipation in PD (NCT03377322).^32^ Besides, leptin as a probiotic substance affects gut microbiota and vagus nerve, which plays an important role in liver and brain function. Leptin action in the liver exerts its anti-steatotic effects and promotes a decreased Firmicutes and an increased Bacteroidetes in the intestine. Leptin action in the CNS also exerts its anti-steatotic effects by increasing hepatic triglyceride secretion and reducing liver de novo lipogenesis (DNL), which requires intact vagal innervation of the liver. In a randomized, placebo-controlled crossover trial, leptin protects from hepatic steatosis independently of food intake by stimulating VLDL secretion and reducing hepatic DNL via a vagal mechanism (EudraCT Nr. 2017-003014-22). Besides, netrin-1 accelerates liver regeneration after partial hepatectomy in mice, and the potential mechanism is related to the promotion of vagus nerve repair and regeneration.^331^ Therefore, leptin targeting the gut-liver-brain axis is supposed to become a promising drug in the future.

In another clinical study, the treatment of *Lactobacillus bulgaricus* and *Streptococcus thermophilus* drastically decreases the levels of ALT, aspartate aminotransferase (AST) and γ-glutamyltransferase in patients with NASH (Table 2).^332^ In addition, some probiotics also display effectiveness for the treatment of persistent gastrointestinal symptoms and depression in patients with IBS^333,334^ neurophysiological patterns in patients with ASD,^335^ and other depressive symptoms.^336,337^

Prebiotics contain no live microbes and nondigestible dietary components that promote the formation of indigenous microbiota in liver and brain diseases.^338,339^ Generally, prebiotics boosts bacterial metabolites of SCFAs, promote the growth of indigenous *Bifidobacteria* and *Lactobacilli* as well as other beneficial bacterial species, lower luminal pH, increase expression of GLP-2,^340^ and prevent pathogen growth and endotoxin transfer in liver disease,^341^ anxiety and depression.^342^ For example, some soluble fibers alter the neuroendocrine stress response and regulate the processing of information that is significantly associated with anxiety and depression.^342^ Oligofructose and inulin-type fructans as common prebiotics boost the abundance of *Bifidobacterium spp* and dramatically reduce liver steatosis and NAS (NCT03184376 and NCT03042494).^343,344^ Meanwhile, galactooligosaccharides reduce the neuroendocrine response to stress and enhance the processing of positive over negative attentional vigilance in patients with stress-related disorders.^33^

Synbiotics as a mixture of prebiotics and probiotics improve multiple gut liver brain axis-related diseases. One symbiotic with 28-week treatment includes 200 million bacteria of seven strains such as *Bifidobacterium breve, Bifidobacterium longum, Lactobacillus acidophilus, Lactobacillus bulgaricus*, *Lactobacillus casei, Lactobacillus rhamnosus*, and *Streptococcus thermophilus*, prebiotics like fructooligosaccharide, and Vitamin A, C and E leads to a substantial decrease of aminotransferases, liver inflammation, and fibrosis formation in patients with NAFLD (NCT01791959)^345^ (Table 2). Meanwhile, the treatment of *Bifidobacterium longum* and fructooligosaccharide significantly reduces hepatic fat formation and the NASH activity index compared with lifestyle modification treated alone.^346^ Besides, some other synbiotics reduce parts of blood lipid markers in patients with obese,^347^ regulate metabolite synthesis and have complex effects on cognitive, affective, and neurological factors associated with health and illness.^348^

### Fecal microbiota transplantation application

Fecal microbiota transplantation (FMT) as a novel strategy to treat gut liver brain axis-related diseases involves transferring gut microbiota from a healthy donor to a damaged recipient. Firstly, the gut microbiota can be rebuilt in liver disease (Table 2).^349^ Two studies (NCT03803540 and NCT02469272), not yet recruiting, are registered to explore the potential advantages of FMT on hepatic histological abnormalities (NCT03803540) and MRI-assessed steatosis (NCT02469272). Although promising, it’s required to further assess FMT treatment on liver histological abnormalities in the early stages of NASH and determine whether it delays NASH development. Secondly, FMT rebuilds a healthy microbial composition and displays positive effects on PD through the gut-brain axis. In rotenone-induced gut dysbiosis, FMT therapy repairs gut microbiota dysbiosis and suppresses inflammation induced by the LPS-TLR4 signaling pathway both in the gut and brain.^350^ Besides, the microbiota from AD mice impairs neurogenesis by increasing colonic inflammation, which contributes to memory loss.^351^ FMT from senescence-resistant mice to AD mice improves spatial learning and memory.^352^

### Other diets application

Polyphenols as plant-derived components are major metabolized by intestinal microbiota in the colon^353^ and benefit in many metabolic-related diseases such as type 2 diabetic,^354^ NASH,^355^ NAFLD,^356^ aging, and so on. Therefore, the high percentage of polyphenols is now recommended by the European Association for the Study of Diabetes, European Association for the Study of Obesity, and European Association for the Study of the Liver guidelines for the people with gut-liver-brain axis-related diseases. For example, cranberry extract reverses the high fat & high sucrose-induced gut microbiota alterations (*Akkermansia spp*.) and improves metabolic syndrome (Table 2).^357^ Green-Mediterranean diet, amplified with polyphenols and unsaturated fat acids, reduces lipid accumulation and improves NAFLD.^358^ Besides, a diet rich in polyphenols reduces liver fat accumulation through the inhibition of de novo lipogenesis (NCT03380416).^359^ Dietary polyphenols, such as isoflavone, lignans, and their metabolites derived from intestinal microorganisms can cross the intestinal barrier and the BBB and prevent neuroinflammatory stimulation.^360^ Besides, tea polyphenol (-)-epigallocatechin-3-gallate weakens the HPA axis, increases the content of SCFAs, regulates gut-brain communication, and alleviates aging impairment.^361^

The low-FODMAP diet including fermented oligosaccharides, monosaccharides, disaccharides, and polyols^362^ is regarded as the first-line therapy for IBS.^363^ It’s used for short-term therapy of certain IBS symptoms but is not utilized as a long-term treatment.^364,365^ Short-term therapy of FODMAP reduces dietary consumption which outperforms antispasmodic medication or moderate FODMAP diet (NCT05182593 and NCT02667184)^366,367^ in alleviating IBS symptoms. Meanwhile, this diet results in less gas and less active microbial metabolite production, which alleviates the sensation of bloating, flatulence, and pain.^368,369^ On the contrary, the compliance of its long-term therapy is poor,^370,371^ which causes a drop in gut microbial diversity and richness, notably of butyrate-producing strains, and has detrimental effects on gut health.^372^

### Nanotechnology application

Nanotechnology is constantly developing and improving in the diagnosis and treatment of gut liver brain axis-related diseases. It can manipulate interactions across microscopic and molecular length scales in the microbiome and has the potential to noninvasive and real-time microorganism intervention technique in gut liver brain axis-related diseases. For example, a gut-liver-axis chip contains the gut epithelial cell chamber and a three-dimensional uniform-sized liver spheroid chamber. Its two chambers are separated by a porous membrane to let the hepatocytes in but inhibit microorganisms entering the chamber.^373^ Nano-poly-boronic acid regulates sugar intake and liver lipogenesis, and finally prevents fructose and glucose absorption in the gut.^374^ In addition, certain microorganisms’ components are prepared into nanotechnology like light-sensitive *Lactococcus lactis* which is an oral live biotherapeutic agent that makes communication from the gut to the host more manageable. This engineered microorganism enhances small intestine targeting and exogenous *Lactococcus lactis* production, allowing for precise regulation of anxiety, vagal afferent and cognitive impairment.^375^ Besides, the honokiol nanoscale drug delivery system also regulates gut microbiome composition and decreases tau hyperphosphorylation, neurological inflammation and Aβ deposition.^376^ Therefore, they are a noninvasive and real-time microorganism intervention technique.

## Therapies targeting gut-liver axis

### Targeting BAs-related pathways

Nowadays, although BAs involved in the pathogenesis of gut-liver and gut-brain axis, targeting BAs-related pathways is only used in liver-related diseases in clinics. BAs metabolism in the gut-liver axis is regulated by two main receptors including FXR and TGR5. FXR activation inhibits BAs production and BAs influx, promotes BAs efflux, and thus alleviates the excessive accumulation of BAs caused by liver disease.^377^ Currently, the most widely used FXR agonists contain primarily BA derivatives, steroidal compounds, and nonsteroidal compounds (Table 2).

As an FXR agonist, UDCA as a primarily BAs derivative, is used to treat cholestatic liver diseases. It has been proposed as a possible treatment for NASH and NAFLD. However, its clinical effectiveness must be validated further.^378^

Obeticholic acid as a steroidal FXR agonist reduces fibrosis and essential NASH features in a phase III trial (NCT02548351).^379^ However, it causes side effects, such as mild to moderate itching, a decrease of high-density lipoprotein-cholesterol (HDL-C), an increase of low-density lipoprotein-cholesterol (LDL-C), and drug-induced hepatotoxicity.^380^ EDP-305, another powerful steroidal FXR agonist, lowers the levels of hepatic ALT and fat in phase IIa clinical trial (NCT03421431). Its adverse events are the same as that of obeticholic acid such as pruritus, nausea, vomiting, diarrhea, headache, and dizziness.^381^

Nonsteroidal FXR agonists are constantly emerging in clinical studies. Among these compounds, cilofexor and TERN-101 (NCT04328077) display positive effects in patients with NASH in phase I and II clinical trials.^382,383^ In a phase I study, although cilofexor has no effect on cholesterol, it dose-dependently reduces the level of FGF19 (NCT02654002). Meanwhile, cilofexor shows well-tolerated and effective in the reduction of hepatic steatosis, liver biochemistry, and serum BAs in a phase II clinical trial (NCT02854605).^382^ However, some of the nonsteroidal FXR agonists display negative and inconclusive effects. For example, nidufexor fails to improve the level of ALT in a phase II clinical trial (NCT02913105). Tropifexor is also terminated in a phase II clinical trial because of its mild pruritus and minor dose-related increase in LDL (NCT02855164). MET409 being evaluated for the treatment of NASH by Metacrine Investigative Site lowers liver content of fat, but still induces differentiated pruritus and LDL-C profile.^384^ The safety and efficacy of EYP001a in patients with NASH are also evaluated in a phase IIa trial (NCT03812029). However, its results are not published up to now.

TGR5 as another BAs receptor exists on the membrane of L cells and influences BAs homeostasis through the gut-liver axis. TGR5 agonists include LCA, DCA, the semi-synthetic BAs, and so on (Table 2).^385,386^ For example, INT-767 and INT-777 as semi-synthetic BAs activate cAMP, stimulate secretion of GLP-1, and improve hepatic glucose and lipid metabolism in NASH/NAFLD.^387^

Omega-3 fatty acid as one kind of N-3 PUFA (omega-3 polyunsaturated fatty acids) lowers liver fat and influences BAs metabolism in a variety of ways.^388^ However, it fails to improve the primary outcome of histological activity in patients with NAFLD (NCT00681408). Now there are another two completed phase II clinical trials without published results involved in Omega-3 fatty acid treatment (NCT01056133 and NCT00845845). Moreover, dietary docosahexaenoic acid as another polyunsaturated fatty acid attenuates blood lipid levels, liver damage and reverses liver metabolism, oxidative stress, and fibrosis formation in NAFLD, which is superior to dietary eicosapentaenoic acid.^389^

### Targeting intestinal mucosa secretions

TGR5 can be stimulated by dietary ingredients and hormonal variables such as insulin and leptin to further trigger the release of gut-derived incretin hormones like GLP-1 (Table 2).^390^ Although gut hormones also involved in the pathogenesis of the gut-liver and gut-brain axis, targeting intestinal mucosa secretions is only used in clinical liver-related diseases. Meanwhile, GLP-1 receptor antagonists such as liraglutide, semaglutide, ALT-801, and dulaglutide promote pancreatic insulin production and inhibit glucagon secretion in protecting against NAFLD development. Endogenous GLP-1 is degraded by dipeptidyl peptidase-4 in minutes. Long-acting human GLP-1 analogs include liraglutide and semaglutide.^391^ Liraglutide displays safe, adequately tolerated, and increased either hepatic and global/localized adipose insulin sensitivity, resulting in lowering the blood quantity of lipotoxic metabolites and inflammatory cytokines in a phase II clinical trial (NCT01237119).^392,393^ Semaglutide possesses a similar mechanism to that of liraglutide and has more dramatic impacts on metabolism and bodyweight reduction in NASH patients than liraglutide does.^394^ However, it is unable to ameliorate the fibrosis stage (NCT02970942).^395,396^ Moreover, microbiota analysis illustrates that GLP-1 receptor antagonists alter the variety of gut microbiota by decreasing the relative abundance of *Proteobacteria* and increasing the relative abundance of *Akkermansia muciniphila*, which are associated with the treatment of NAFLD.^397^

FGF19 is released by intestinal cells of the terminal ileum following FXR activation by BAs. FGF19 flows from the intestine into the liver through portal vein circulation and combines with FGFR4 and β-klotho to reduce the production of BAs. FGF19 analogs (NGM282/Aldafermin) modulate BAs production, lipid metabolism, and gluconeogenesis. BAs-activated FXR increases FGF19 gene expression and production.^398^ NGM282 demonstrates an adequate safety profile in patients with NASH in a 12-week phase IIa trial. It decreases the levels of liver fat, and improves NAS, fibrosis scores, and other liver function indicators (NCT02443116).^399^ Further stage II research with patients with NASH is active but not recruiting (NCT03912532).

### Novel therapeutic applications

#### Intestinal permeability and microbiota-targeting therapy

Claudins, especially claudin-2 plays a crucial role in the formation of gated paracellular channel and regulation of TJs channels, which may be ideal therapeutic targets for affecting the epithelial barrier and improving intestinal permeability.^400^ Importantly, occludin S408 dephosphorylation regulates TJs channel gating dynamics and protein molecule interaction, which have therapeutic significance for inflammation-related intestinal barrier disorders.^401^ However, more clinical studies are needed to determine pharmacological methods for regulating gating activity for therapeutic purposes. In addition, microRNA-155 regulates NF-κB signal, reduces expression of TNF-α, IL-6, ZO-1, and occludin, and then inhibits inflammation and intestinal barrier dysfunction in mice.^402^

Non-selective beta blockers (NSBBs) play important roles in the management of portal hypertension in liver cirrhosis during the last three decades. NSBBs increase levels of intestinal permeability and bacterial translocation indicators like IL-6/LPS binding proteins.^403^ They improve intestinal hypomotility in the setting of sympathetic activity, minimize the overgrowth development of small intestine bacteria and are associated with a lower incidence of spontaneous bacterial peritonitis in cirrhosis.^404^

Bacteriophages as viruses are constantly developed to selectively infect and destroy the defensive system of bacteria.^405^ Because bacteriophages operate in an entirely orthogonal action mode in comparison with antibiotics, they do not meet their resistance, and even effectively destroy the extremely antibiotic-resistant bacteria.^406^ Therapeutic bacteriophages delivery under compassionate procedures has antibacterial effect in terminally ill patients with *Enterococcus faecium, Staphylococcus aureus, Klebsiella pneumoniae, Acinetobacter baumannii, Pseudomonas aeruginosa*, and *Enterobacter species* infections.^407,408^ Therefore, many biotechnological corporations pay attention to this technology and plan to convert it into clinical practice.^409^

#### LPS-targeting therapy

High density lipoprotein (HDL) is synthesized in the liver and small intestine where its core structural protein, apolipoprotein A1 (apoA1) is synthesized. HDL removes endotoxins, prevents infection,^410^ neutralizes LPS, and speeds LPS clearance in mice via SR-BI (scavenger receptor class B, type I)-mediated LPS absorption. LPS attaching to apolipoprotein AI or apolipoprotein E inhibits inflammatory response, while apolipoprotein AII or apolipoprotein CI binding to LPS promotes inflammation. Besides, intestine-derived HDL_3_ has a unique role in the prevention of liver damage from gut-derived LPS, implying that HDL_3_ might be a target for treating liver illness linked with gut leakiness.^411^

Yaq-001 as a newly synthesized non-absorbable carbon has a high adsorption capacity for endotoxin and LPS. Yaq-001 treatment increases the composition and function of the microbiome, as well as the function of circulating innate immune cells in rats with bile duct ligation. Meanwhile, it is regarded as a new strategy to resist the change of gut microbiota and bacterial product translocation in patients with advanced liver disease.^412^ Therefore, it is mostly given orally in tiny bags and tested in clinical research on decompensated liver cirrhosis (NCT03202498).

## Therapies targeting the gut-brain axis

Some therapeutic measures play a special role in gut-brain axis metabolism, such as cognitive behavioral therapy (CBT), antidepressants, and therapies targeting tryptophan metabolism.

### Cognitive behavioral therapy

CBT as a viable therapeutic option for IBS symptoms^413^ reduces anxiety, stimulates health-promoting habits, takes greater responsibility, controls over their treatment and enhances pain tolerance.^414^ Recent research assesses the effectiveness of clinic-based CBT, home-based CBT, and IBS education for treating IBS symptoms, quality of life changes in feces consistency, emotional distress, and satisfaction with treatment (Table 3).^415^ As 12-month clinical trial indicates, both clinic-based or home-based CBT reduce IBS symptoms, while IBS education does not display these benefit.^416^ Patients who received home-based CBT are more likely to persevere owing to their self-monitoring, self-learning and correction of inaccurate threat assessments by imbalanced gut-brain relations.^416,417^ Furthermore, CBT reduces inappropriate illness cognitions, triggers shift in self-processing, decreases biases in self-referent illness and health processing, and enhances awareness without judgment in patients with IBS (NCT02794376).^418^

**Table 3:** Clinical trials targeting gut-brain axis

Classification	Formula	Diseases	Study status	NCT number
Low FODMAP diet	Fermentable oligosaccharides, disaccharides, monosaccharides, and polyols	Abdominal Pain	Unknown status^456^	NCT04528914
		IBS	Positive^457^	NCT03678935
			Positive^363^	NCT05182593
			Positive^458^	NCT04283487
			Positive^459^	NCT02980406
			Positive^369^	NCT02667184
			Positive^460^	NCT02107625
			Positive^461^	NCT03304041
			Positive^462^	NCT02161120
			Positive^463^	NCT02210572
			Positive^464^	NCT03586622
			Positive^465^	NCT04256551
			Positive^466^	NCT03653689
			Positive^467^	NCT03268720
			Positive^468^	NCT04072991
			Negative^469^	NCT04296552
			Positive^470^	NCT04270487
		Fecal incontinence	Positive^471^	NCT02828384
		Fibromyalgia	Positive^472^	NCT04007705
Probiotics	Vivomixx® *Bifidobacterium infantisBi-26, Lactobacillus* *rhamnosusHN001, Bifidobacterium lactisBL-04* and *Lactobacillus* *paracaseiLPC-37*	ASD	Positive^473^	NA
	*Streptococcus thermophilus* DSM 24731, *Bifidobacterium* (B. breve DSM 24732, B. longum DSM 24736, B. infantis DSM 24737), *Lactobacillus* (L. acidophilus DSM 24735, L. plantarum DSM 24730, L. paracasei DSM 24733, L. delbrueckii subsp. bulgaricus DSM 24734)		Positive^335^	NCT02708901
	*Bifidobacterium longum NCC3001*	IBS	Positive^474^	NCT01276626
	*Escherichia coli (DSM 17252)* and *Enterococcus faecalis (DSM 16440)*		Negative^475^	2012-002741-38
	*Bifidobacterium bifidum MIMBb75*		Positive^476^	ISRCTN14066467
	Vivomixx®	Depression	Positive^336^	NCT02957591
	*Bifidobacterium* breve CCFM1025		Positive^337^	ChiCTR2100046321
	*Lactobacillus acidophilus, Bifidobacterium bifidum, Lactobacillus reuteri* and *Lactobacillus fermentum*	Schizophrenia	Positive^477^	IRCT2017072333551N2
			Positive ^477,478^	NA
	*Lactobacillus* plantarum PS128	PD	Positive^479^	NCT04389762
	*Lactobacillus sp* and *Bifidobacterium sp*		Positive^480^	NA
	*Lactobacillus* *acidophilus, Lactobacillus reuteri, Lactobacillus gasseri, Lactobacillus rhamnosus*, *Bifidobacterium bifidum, Bifidobacterium longum, Enterococcus faecalis* and *Enterococcus faecium*		Positive^32^	NCT03377322
	*Streptococcus salivarius subsp thermophilus, Enterococcus faecium, Lactobacillus rhamnosus GG, Lactobacillus acidophilus, Lactobacillus plantarum, Lactobacillus paracasei, Lactobacillus delbrueckii subsp bulgaricus* and *Bifidobacterium* *(breve* and *animalis subsp lactis)*		Positive^481^	NCT02459717
	*Lactobacillus acidophilus, Bifidobacterium bifidum, Lactobacillus reuteri* and *Lactobacillus fermentum*		Positive^482^	IRCT2017082434497N4
	*Lactobacillus acidophilus*, *Lactobacillus plantarum, Lactobacillus casei*, *Lactobacillus helveticus, Lactobacillus brevis*, *Bifidobacterium lactis, Bifidobacterium lactis* and *Streptococcus salivarius subsp. Thermophilus*	Epilepsy	Positive^483^	NCT03403907
	*Lactobacillus casei, Lactobacillus* *acidophilus, Lactobacillus rhamnosus, Lactobacillus* *helveticus, Lactobacillus bulgaricus, Lactobacillus* *plantarum, Lactobacillus gasseri, Bifidobacterium* *breve, Bifidobacterium longum, Bifidobacterium* *lactis, Bifidobacterium bifidum, and Streptococcus* *thermophilus*, and *fructooligosaccharides*	Migraine	Positive^484^	NA
	*Bacillus subtilis PXN 21*, *Bifidobacterium bifidum PXN 23, Bifidobacterium* *breve PXN 25, Bifidobacterium infantis PXN 27, Bifidobacterium longum PXN 30, Lactobacillus acidophilus PXN 35, Lactobacillus delbrueckii ssp. bulgaricus PXN 39, Lactobacillus casei PXN 37, Lactobacillus plantarum PXN 47, Lactobacillus rhamnosus PXN 54, Lactobacillus helveticus PXN 45, Lactobacillus salivarius PXN 57, Lactococcus lactis ssp. lactis PXN 63* and *Streptococcus* *thermophilus PXN 66*		Positive^485^	NA
	*Bifidobacterium bifidum W23*, *Bifidobacterium lactis W52, Lactobacillus* *acidophilus W37, Lactobacillus brevis W63*, *Lactobacillus casei W56, Lactobacillus salivarius* *W24, Lactococcus lactis W19* and *Lactococcus* *lactis W58*		Negative^486^	NA
CBT	10 sessions of clinic-based CBT or 4 sessions of largely home-based CBT with minimal therapist contact over a 10-week acute phase	IBS	Positive^487,488^	NCT00738920
			Positive^418^	NCT02794376

Clinical study status is classified into seven categories: positive, negative, recruiting, not recruiting, completed, terminated, withdrawn, and unknown. Positive indicates the primary endpoint has been reached. Negative indicates that the primary endpoint was not met or that there were substantial adverse effects in the trial. The data is available in Clinical Trials (https://www.clinicaltrials.gov)

### Novel therapeutic applications

#### Antidepressants application

Antidepressants as neuromodulators at low doses regulate brain-gut communication. Treating inflammatory bowel disease (IBD) involves in activation of vagus nerve-mediated anti-inflammatory capabilities and direct influences on pro-inflammatory cytokines. The primary impact on cytokines depends on the NF-κB and nitric oxide pathway, both of which promote IBD development.^419^ In a multicenter clinical trial, 5-hydroxytryptophan as one kind of antidepressant significantly regulates depression, anxiety, and stress scores, but not improves IBD-related fatigue (NCT03574948).^420^

#### Targeting tryptophan metabolism application

The microbiota has a direct or indirect influence on the three primary tryptophan metabolites including serotonin, kynurenine, and indole derivatives in the gut,^421^ which is associated with intestinal inflammation and IBD. Reintroducing xanthurenic and kynurenic exerts a defensive impact via reorganizing energy metabolism in aryl hydrocarbon receptors, intestinal epithelial cells, and CD4^+^ T cells. Therefore, targeting tryptophan metabolism repairs abnormalities in endogenous metabolic pathways in IBD.^422^

## Therapies targeting the liver-brain axis

### Targeting epigenetic regulation

Epigenetic regulation in the liver-brain axis, like RNA methylation, should be mentioned in the treatment of liver and brain-related diseases.^423^ Exercise-mediated restoration of m6A methylation in the mouse medial prefrontal cortex, whose activity is potentiated to produce anxiolytic effects, is demonstrated by a combination of molecular, behavioral, and in vivo recording data. Furthermore, it demonstrates that hepatic manufacture of one methyl donor is required for exercise to enhance brain RNA m6A methylation in order to offset environmental stress. Through the liver-brain axis, exercise training brings fresh insights into the diagnosis and treatment of anxiety disorders.^286^

### Targeting Aβ metabolism

Hepatic soluble epoxide hydrolase targets Aβ metabolism and significantly treats neurological diseases in the liver-brain axis. It bidirectionally regulates the plasma levels of 14,15-epoxyeicosatrienoic acid, which rapidly crosses the BBB and modulates brain Aβ metabolism via multiple pathways. Therefore, hepatic soluble epoxide hydrolase attenuates brain burden, tauopathy, and cognitive deficits, which may be a potential treatment method for liver-brain diseases.^424^

## Future perspective

In the past decades, gut-liver-brain axis communication has become an important research topic.^25,425^ Researchers make efforts to analyze their communication mechanism among all nodes in the axis.^4,426^ Now, it is widely accepted that the gut, liver, and brain maintain a stable balance is beneficial for multiple disease progression.^427–429^

The disorder of the gut-liver-brain axis influences disease development and progression, which includes the change of intestinal permeability, SCFAs, FIAF, choline, ethanol, BAs metabolism, BBB, vagus nerve, neurotransmitters, gut hormones, microbial metabolites, immunity, ammonia, lactate and manganese accumulation, epigenetic regulation and Aβ metabolism. Clinical trials using antibiotics, probiotics, prebiotics, synbiotics, FMT, polyphenols, and novel nanotechnology confirm the critical role of the gut-liver-brain axis in different diseases. Besides, some special treatments such as FXR agonists, TGR5 agonists, GLP-1 receptor antagonists, FGF19 analogs have a beneficial effect on the maintenance of gut-liver axis balance. Most importantly, FXR agonists and probiotics enters phase III clinical studies, which increased some bacteria such as *Bifidobacterium* and *Lactobacilli*, and displays positive therapeutic effects. The emergence of some promising therapies including intestinal permeability and microbiota and LPS-targeted treatment like claudins, NSBBs, bacteriophages, HDL and Yaq-001 also provides impetus for future multiple liver disease treatments. Moreover, some special therapies regulating the gut-brain axis include CBT, antidepressants and tryptophan metabolism targeted therapy, which also display positive effects in brain diseases. So far, low FODMAP diets and CBT have entered the phase IV and III clinical studies. They exhibit useful for short-term treatment of certain IBS symptoms in multiple clinical trials. Furthermore, emerging manipulations such as antidepressants and tryptophan metabolism targeted therapies may be providing many opportunities for the treatment of gut-brain axis-related diseases. In addition, some promising therapies targeting the liver-brain axis include targeting epigenetic regulation and Aβ metabolism, which opens a new path for future development.

In conclusion, the gut liver brain axis plays an indelible role in influencing multiple diseases. According to the summary of these mechanisms, some metabolites including LPS, SCFAs, BAs, immunity, some inflammatory cytokines and beneficial bacteria such as *Bifidobacterium* and *Lactobacillus* significantly influence the pathogenesis of gut liver brain axis-related diseases. So far, high-quality preclinical research and some randomized controlled trials have demonstrated the effectiveness of some therapies based on the theory of gut liver brain axis. It is expected to further develop more clinical candidates to regulate the gut liver brain axis and treat their related diseases.
